# Joint Frailty Mixture Cure Model for Recurrent Event Data With Dependent Censoring: An MCEM Approach

**DOI:** 10.1002/sim.70579

**Published:** 2026-05-08

**Authors:** Nasrin Sultana, Moudud Alam, Md Hasinur Rahaman Khan

**Affiliations:** ^1^ Applied Statistics and Data Science Institute of Statistical Research and Training, University of Dhaka Dhaka Bangladesh; ^2^ Department of Agricultural and Applied Statistics Gazipur Agricultural University Gazipur Bangladesh; ^3^ School of Information and Engineering Dalarna University Falun Sweden

**Keywords:** complementary log–log, cure fraction, dependent censoring, joint frailty, logistic, recurrence

## Abstract

Advancements in modern medical technology have enabled cures for a fraction of patients while extending survival times for those who are not cured. For non‐cured patients, disease recurrence is influenced by observed covariates and unobserved individual heterogeneity (random effects). In biomedical studies, dependent censoring is frequently encountered, for example, in cancer patients, where right censoring can be caused by death from unrelated diseases or due to an (unobservable) cure status. This study introduces a joint frailty model for recurrent event data with a cure fraction, effectively capturing heterogeneity and inducing dependent censoring. The proposed multivariate joint frailty mixture cure models incorporate covariates and frailties, together with the event incidence time and latent cure status. The model accounts for the probability of a cure after each recurrence using both the complementary log–log and the logistic link function. A likelihood‐based estimation method is developed using the Monte Carlo Expectation‐Maximization (MCEM) algorithm. Through Monte Carlo simulation, we examine the finite sample properties of the MCEM estimators, supplemented by a real‐world application using secondary data on hospital readmissions for colorectal cancer recurrence post‐surgery. Simulation results suggest lifetime and frailty parameter estimates are unbiased and consistent. Compared to models with identical frailty structure, both the complementary log–log and the logistic cure frailty models with dependent frailties demonstrate a better fit with the real data, as evidenced by lower Akaike information criteria values.

## 1 Introduction

Repeated occurrence of an event, or recurrence, is frequently observed in biomedical studies. For example, cancer patients often experience conditions such as multiple tumor recurrences after breast cancer surgery or undetected cancer cells which remain after treatment. Even so, the immune system or resistance of patients may deteriorate after surgery leading to the prompt spread of cancer throughout the body. Repeated heart attacks in patients with cardiovascular disease, psychological disorders such as depression or anxiety, and tuberculosis are also examples of recurring events in lifetime data. Multiple failures of a machine after repair and recurrent downtime of a server are examples of recurrent events in reliability engineering. Analysis of these recurring‐event data is important to address some specific queries regarding the comparative study of new treatments/therapies or to examine the chance of recovery within the disease evolution process [1, 2, 3].

In survival analysis, it is commonly assumed that all patients will eventually experience the event of interest. However, in reality, not all the individuals in the study are at risk of the event during the entire study period, even if the study period is extended. These fractions of long‐time survivors are known as cured. Modern developed medical technology and treatments, for example, early‐stage diagnosis or effective therapies within different types of oncology studies such as breast cancer, leukemia and melanoma, or prostate cancer, today give rise to the mixture cure models which are important and popular in clinical trials and medical research [3, 4, 5]. These mixture cure models are suitable for the precise estimation of the survival time and the probability of a subject to be cured with covariate effects.

A dependent censoring mechanism is observed when the censoring time is associated with the likelihood of being cured, or the event occurrence time among non‐cured individuals [4]. For example, in the case of cancer studies (prostate, colorectal, or breast), some patients may be considered as cured (censored) with respect to the terminal event being death due to cancer while non‐cured cancer patients are less likely to be censored, leading to dependent censoring. In the dependent censoring mechanisms, frailty models have been studied to determine the dependence between failure and censoring [6, 7, 8, 9].

For recurrent event times, the frailty models have been widely utilized to model unobservable random effects or frailty [1, 2, 10, 11, 12]. Recently, many studies have extended these models associated with cure fractions for individual data, especially when analyzing cancer data [13, 14, 15, 16, 17, 18, 19]. Previously, a single frailty term was used as a multiplicative component in joint frailty models to capture unobserved heterogeneity due to latent risk factors beyond covariates. In a cure mixture model for recurrent‐event data, time to event was observed only for the non‐cured subjects, meaning the individuals with a recurrence cannot belong to the cured group [20, 21]. The probability of incidence of an event can be associated with some influential observed and unobserved factors, where ignoring these random effects may produce biased estimates. Moreover, after each recurrence, it is necessary to estimate the incidence probability to capture the instantaneous likelihood of future recurrence. Patients may improve the risk of recurrence or may deteriorate due to medicine reactions after readmission. Inclusion of a new frailty after each recurrence may address this individual‐level heterogeneity [22]. Logistic, probit, or complementary log–log link functions are suitable for modeling cure probability as they offer flexibility when capturing changes after each recurrent [21].

Rondeau et al. [5] modeled recurrent events using frailty model with a cured fraction after each recurrence, where shared random effects were adopted and proportionally connected between the recurrence and survival components. In reality, distinct yet correlated frailty terms for latency (or cure probability) and incidence (or time to event) parts may be a better choice to capture the underlying dependence or joint heterogeneity between these processes, which was not explored in their study. Convergence failure is commonly observed with numerical optimization of the likelihood function for models with multiple or non‐normal random effects, high‐dimensional mixture distributions, or complex hierarchical models [23]. Relying on the normality assumption of the frailty distribution, PROC NLMIXED in SAS 9.1 and Gaussian quadrature were used to maximize the likelihood function in the study by Rondeau et al. [5]. Although PROC NLMIXED is a flexible and powerful tool for the estimation of parameters in nonlinear mixed‐effect models, it also has several constraints. The accuracy of numerical integration when calculating the marginalized likelihood depends on the number of quadrature points as well as the dimensionality of the random effects [24]. Rondeau et al. [5] also reported convergence instability up to 4% for the simulated cases in their study. The baseline hazard is generally modeled under parametric assumptions such as piecewise constant or Weibull which may fail to model complex or non‐proportional hazard patterns. However, this model produced a biased estimate of the variance of the random effects as well as bias in the regression parameter estimates, and the bias of the estimates was not reported across different cure fractions.

Tawiah et al. [3] proposed a more general bivariate joint frailty mixture cure model with correlated random effects. This model jointly accounts for the dependence between the hazard rates of recurrent events and death among non‐cured patients. The probability of being cured was modeled using a logistic function which included patient‐specific unobserved frailty, uncorrelated with other frailties, although this failed to capture the correlation between event time and cure status. The proposed Expectation and Maximization (EM) type restricted maximum likelihood (REML) estimation method, when contrasted with the Markov Chain Monte Carlo (MCMC), is a less time‐consuming process. However, this algorithm replaces the missing cure status with their conditional expectations, given frailties, and produced biased parameter estimates depending on the underlying correlation pattern.

This study aims to develop a flexible joint frailty model where two dependent random effects, one for incidence and the other for hazard, are considered separately to obtain a smooth underlying model structure. Both the complementary log–log link function and the logistic link function were applied to model the non‐cured status of the patients. The complementary log–log is suitable when estimating the probability of rare events or events with rapidly increasing risk, while the logistic link function can handle symmetric connections effectively. The performances of the proposed models were investigated through a series of simulation studies. Parameter estimation and inference were conducted using the Monte Carlo (MC) Expectation‐Maximization (MCEM) algorithm. In addition, the usefulness of the proposed procedures was exemplified by analyzing secondary data on successive rehospitalization of patients diagnosed with colorectal cancer published by González et al. [25].

The remainder of this article is structured as follows. Section 2 presents the proposed mixture cure frailty model formulation for recurrent events. The likelihood function and parameter estimation process are also explained in detail in this Section. Section 3 explains the simulation settings and discusses the simulation results. The performance of the proposed models is further evaluated using real data on the successive rehospitalization of patients diagnosed with colorectal cancer, with comparisons made against a mixture cure model incorporating identical frailty discussed in Section 4. A concluding discussion of the merits and limitations of the proposed models and their estimation technique is presented in Section 5.

## 2 Methods

### 2.1 Proposed Mixture Cure Frailty Model for Recurrent Events

Suppose a longitudinal follow‐up study is conducted to observe the recurrent clinical event of N independent patients. Right censoring may occur in the recurrent event process subject to death, loss of follow‐up, or end of the study (i.e., administrative censoring). Let $X_{ij}$ be the jth ($j=1,2,\ldots ,n_{i}$) recurrent time of ith (i=1,2,…,N) individual, $C_{ij}$ is the censoring time for jth recurrence of ith individual and $T_{ij}=min\left(X_{ij},C_{ij}\right)$ corresponds to each follow‐up time. Let $\delta_{ij}$ be the censoring indicator for ith individual at jth recurrence, then $\delta_{ij}=I\left(X_{ij}<C_{ij}\right)$ can be set to 0 for censored, and 1 otherwise. Let $Z_{ij}=\left(z_{1ij},z_{2ij},\ldots ,z_{pij}\right)$ be a fixed or time‐dependent observed covariate vector of dimension p associated with survival time for jth recurrence of ith individual. Suppose, $\omega_{1i}$ is a random effect, or frailty, with probability density function $g\left(\omega_{1}\right)$ where $\omega_{1}=\left(\omega_{11},\omega_{12},\ldots ,\omega_{1N}\right)$, capturing the dependence of an individual being successively readmitted due to events. Distinct genetic variants, hormones, susceptibility to carcinogens, or any other particular unobserved factors can be the sources of correlation between successive recurrences within each patient.

The Cox proportional hazard model allows frailty in the model to capture the unobserved heterogeneity and is expected to induce the dependence among recurrent events within the same individual [10]. However, Cox's model [26] also assumes that all individuals experience the event, which is not true in practice since all the individuals in the study may not experience the event or may not even be at risk of developing disease during the study period or later in life. Therefore, the idea of extending the ordinary frailty model to cure frailty is motivated by the need to model the susceptible and non‐susceptible individuals separately [27]. To identify the cure status of an individual after each recurrent visit, let $U_{ij}=1$ denote the individual experiencing the event (non‐cured, at jth incidence) and $U_{ij}=0$ to denote the opposite (cured).

The patients who successfully responded to treatment and are no longer at risk for recurrence might be more likely to drop out of the study. In contrast, the patients who are still at risk for recurrence are more likely to remain in the study and less likely to drop out prematurely. Consequently, such dependence on the censoring mechanism is evident in the cure fraction model. The hazard for a non‐cured individual at time $t_{ij}$ given $Z_{ij}$ and a random subject‐specific frailty $\omega_{1i}$ is given by

(1) $$\lambda_{ij}\left(t_{ij}|U_{ij}=1,\omega_{1i}\right)=\lambda_{0}\left(t_{ij}\right)\exp\left[Z_{ij}^{T}\beta +\omega_{1i}\right],$$

where $\omega_{1i}⊥\omega_{1i^{\prime }}\forall i\neq i^{\prime }$, $\lambda_{0}$ is the so‐called baseline hazard which does not involve β or $\omega_{1}$, and β is a p‐vector of unknown parameters. The survival function corresponding to Equation (1) is given by

(2) $$S\left(t_{ij}|U_{ij}=1,\omega_{1i}\right)=\exp\left[-\Lambda_{0}\left(t_{ij}\right)\exp\left[Z_{ij}^{T}\beta +\omega_{1i}\right]\right],$$

where $\Lambda_{0}\left(t_{ij}\right)=\int_{0}^{t_{ij}}\lambda_{0}\left(t\right)dt$, is the cumulative baseline hazard function, specified as non‐parametric. The associated density function of Equation (2) is given by

(3) $$\begin{array}{cc} f\left(t_{ij}|U_{ij}=1,\omega_{1i}\right) & =\exp\left[-\Lambda_{0}\left(t_{ij}\right)\exp\left[Z_{ij}^{T}\beta +\omega_{1i}\right]\right]\lambda_{0}\left(t_{ij}\right)\\ & \;\times \exp\left[Z_{ij}^{T}\beta +\omega_{1i}\right].\\ & =S\left(t_{ij}|U_{ij}=1,\omega_{1i}\right)\lambda_{ij}\left(t_{ij}|U_{ij}=1,\omega_{1i}\right). \end{array}$$

In this study, the mixture cure model with different cure status is considered to make a relative comparison. The first model uses a complementary log–log (or clog–log) model for the cure status

(4) $$P\left(U_{ij}=1|\omega_{2i}\right)=\exp\left[-\exp\left[V_{ij}^{T}\gamma +\omega_{2i}\right]\right]=\pi_{ij}\left(U|\omega_{2i}\right).$$

while the second model is designed with a logistic cure status

(5) $$P\left(U_{ij}=1|\omega_{2i}\right)=\frac{\exp\left[V_{ij}^{T}\gamma +\omega_{2i}\right]}{1+\exp\left[V_{ij}^{T}\gamma +\omega_{2i}\right]}=\pi_{ij}\left(U|\omega_{2i}\right),$$

where $V_{ij}=\left(v_{1ij},v_{2ij},\ldots ,v_{qij}\right)$ is the observed covariate of dimension q associated with cure probability for jth recurrent of ith individual, γ is q‐vector of parameter, $\omega_{2i}$ is the random effect of ith individual associated with the probability of cure, and $\pi_{ij}\left(U|\omega_{2i}\right)$ is the probability that no additional event occurs after each event.

The frailty $\omega_{1i}$ captures the unobserved heterogeneity in time‐to‐recurrence of ith individual and $\omega_{2i}$ captures the unobserved heterogeneity in the cure probability. Practically, these two random effects are not independent. Individuals with larger $\omega_{1i}$, indicates higher frailty for recurrence. However, smaller $\omega_{2i}$ tends to be less likely to be non‐cured as it is related with increased susceptibility of being cured. Therefore, the correlation of these two random effects is useful for the within‐cluster correlation [5]. The joint density of $\omega_{1i}$ and $,\omega_{2i}$ can be expressed in vector notation $\omega_{i}={\left(\omega_{1},\omega_{2}\right)}^{T}∼f\left(\omega_{i}\right)$ for some density f with marginals $g\left(\omega_{1}\right)$, and $h\left(\omega_{2}\right)$. For $f\neq h\left(\omega_{1}\right)g\left(\omega_{2}\right)$, the model ensures a dependence between the recurrence hazards and the cure probability. However, the frailties, $\omega_{1i}$ and $\omega_{2i}$, are non‐identical; in other words, none of the frailty terms is normally distributed or proportional to each other, which contrasts with other existing works [3, 5]. Distributional assumptions, together with the estimation procedure, are elaborately discussed in the following subsection. The overall survival function, marginal on U, is given by

(6) $$S\left(t_{ij}|\omega_{i}\right)=1-\pi_{ij}+\pi_{ij}\exp\left[-\Lambda_{0}\left(t_{ij}\right)\exp\left[Z_{ij}^{T}\beta +\omega_{1i}\right]\right].$$

The corresponding density function is given by

(7) $$\begin{array}{cc} f\left(t_{ij}|\omega_{i}\right) & =\pi_{ij}\exp\left[-\Lambda_{0}\left(t_{ij}\right)\exp\left[Z_{ij}^{T}\beta +\omega_{1i}\right]\right]\\ & \;\lambda_{0}\left(t_{ij}\right)\exp\left[Z_{ij}^{T}\beta +\omega_{1i}\right]. \end{array}$$

where the recurrence times $t_{i1},t_{i2},\ldots ,t_{in_{i}}$, are the gap times or differences between the successive visits due to the event for the same subject and are independent given the frailty $\omega_{1i}$ and $\omega_{2i}$. A positive value of β indicates an increased risk of recurrence due to an event (if susceptible), while a positive γ suggests that the probability of being cured is lower (or the probability of being susceptible is higher). Throughout the article, vectors and matrices are denoted by bold symbols where the design matrices are bold‐capital, for example, $Z_{\left(N\times p\right)}$ and $V_{\left(N\times q-1\right)}$.

#### 2.1.1 Estimation Procedure for Complementary log–log Model

The gamma distribution is frequently used as a frailty distribution due to its infinitely divisible property [12] which allows the total frailty to be expressed as the sum of many small, independent risk components arising from multiple sources within an individual. Moreover, the gamma frailty model exhibits a time‐invariant dependence structure between frailties in shared frailty settings, offering a balance between interpretability and computational tractability [28]. Several correlated gamma frailty models have been proposed in the bivariate context in the literature [28]; however, in this study, we propose a novel bivariate gamma frailty model by assuming $\omega_{1i}=\log\left(u_{1i}\right)$ and $\omega_{2i}=\log\left(u_{2i}\right)$. Log‐transformed frailties ensure a positivity and an additive effect on the log hazard model as well as in the cure model. The advantages of such parametrization, in additions to its mathematical simplicity, is discussed elsewhere in the literature [29]. To complete the model formulation, let us assume two independent random variables x and y, where x∼Beta(α,α) and y∼Γ(2α,α). Then the product of gamma and beta variables, $u_{1}=xy$ also follows gamma with parameters (α,α) and $u_{2}=2y∼\Gamma (2\alpha ,2\alpha )$ [30]. Therefore, $u_{1i}$ and $u_{2i}$ jointly follow bivariate gamma with the probability density function

$$\begin{array}{l} f\left(u_{1i},u_{2i}\right)=\frac{\alpha^{2\alpha }u_{1i}^{\left(\alpha -1\right)}{\left(u_{2i}-2u_{1i}\right)}^{\left(\alpha -1\right)}\exp\left[-\frac{u_{2i}\alpha }{2}\right]}{2^{\alpha }\Gamma \left(\alpha \right)\Gamma \left(\alpha \right)}, \end{array}$$

where $0<u_{1i}<u_{2i}<\infty$; which implies dependence due to support restrictions $u_{2i}\geq 2u_{1i}$. Furthermore, $u_{2i}$ is a gamma frailty, as well as the random effect in the cure fraction, while $\omega_{2i}$ adds linearly to the respective linear predictor without any range constraints. Here, $E\left(u_{2i}\right)=1$, and $E\left(u_{1i}\right)=1$ which helps to estimate the parameter and improve interpretability [29]. Furthermore, $u_{1i}⊥̸u_{2i}$ implies linear correlation $\mathrm{Corr}(\omega_{1},\omega_{2})=\frac{\psi^{\prime }(2\alpha )}{\sqrt{\psi^{\prime }(\alpha )\psi^{\prime }(2\alpha )}}.$ Positive trigamma in the correlation function always estimates positive correlation as α is positive. In addition, $V\left(u_{1i}\right)=\frac{1}{\alpha }$ and $V\left(u_{2i}\right)=\frac{1}{2\alpha }$ shows only α controls the degree of unobserved heterogeneity; interpreted as α→∞, the variance →0, refers homogeneous population. The complete data likelihood can be expressed in the following form

$$L_{c}=\overset{N}{\underset{i=1}{\prod }}\left(\overset{n_{i}}{\underset{j=1}{\prod }}{\left(1-\pi_{ij}\right)}^{1-U_{ij}}\pi_{ij}^{U_{ij}}G_{ij}\right)f\left(\omega_{i}\right),$$

where

$$G_{ij}={\left[f\left(t_{ij}|\omega_{1i}\right)\right]}^{\delta_{ij}}{\left[S\left(t_{ij}|\omega_{i}\right)\right]}^{\left(1-\delta_{ij}\right)}.$$

If an individual is not cured during the follow‐up time, the censoring indicator, $\delta_{ij}$ is set to 1, otherwise 0. After log transformation the likelihood becomes

(8) $$\begin{array}{cc} l_{c} & =\overset{N}{\underset{i=1}{\sum }}\overset{n_{i}}{\underset{j=1}{\sum }}\left(1-U_{ij}\right)\log\left(1-\pi_{ij}\right)+\overset{N}{\underset{i=1}{\sum }}\overset{n_{i}}{\underset{j=1}{\sum }}U_{ij}\log\pi_{ij}\\ & \;+\overset{N}{\underset{i=1}{\sum }}\overset{n_{i}}{\underset{j=1}{\sum }}\log G_{ij}+\overset{N}{\underset{i=1}{\sum }}\log f\left(\omega_{i}\right). \end{array}$$

The log‐likelihood (Equation 8) can be partitioned into three distinct parts. The first part of likelihood, $l_{\gamma }$, is the contribution based on the parameters of the complementary log–log model (see Equation 9), the second part is associated with the model of mixture cure frailties (see Equation 10), and the third part, $l_{\alpha }=\sum_{i=1}^{N}\log f\left(\omega_{i}\right)$, corresponds to frailties.

An observed individual who experiences a recurrence is defined as non‐cured, that is, $\delta_{ij}=1$ implies $U_{ij}=1$. In contrast, the cure status of an individual is unknown if the respective follow up time ends before a recurrence, that is, $U_{ij}$ is missing when $\delta_{ij}=0$. To overcome this situation, a complete data likelihood is used to deal with both the unobserved and observed cases. The E‐step completes the missing cure status and frailties by estimating the expectation of the complete‐data log‐likelihood given the observed data and the current parameters of $\left(\beta ,\gamma ,\alpha \right)=\left(\beta^{0},\gamma^{0},\alpha^{0}\right)=\theta^{0}$. The M‐step maximizes the conditional expectation of the complete‐data log‐likelihood over the unknown parameters to update the parameter estimate. Iteration between E‐step, and M‐step continues until convergence.

An analytical solution of the expectation of $l_{\gamma }$ is not possible. The EM algorithm can be performed effectively using Monte Carlo EM where in each iteration, total K samples of $u_{2}^{1},u_{2}^{2},\ldots ,u_{2}^{K}$ are generated from the density of $f\left(u_{2i}|u_{1i},t_{ij};\theta^{0}\right)$ and the expectation in $l_{\gamma }$ can be approximated by

(9) $$\begin{array}{cc} 𝔼\left[l_{\gamma }^{k}\right] & =\frac{1}{K}\overset{K}{\underset{k=1}{\sum }}\left[\overset{N}{\underset{i}{\sum }}\overset{n_{i}}{\underset{j}{\sum }}\left(1-U_{ij}\right)\log\left(1-\pi_{ijk}\right)\right.\\ & \;+\overset{N}{\underset{i}{\sum }}\overset{n_{i}}{\underset{j}{\sum }}U_{ij}\log\pi_{ijk}+\overset{N}{\underset{i}{\sum }}\overset{n_{i}}{\underset{j}{\sum }}\delta_{ij}\log\pi_{ijk}\\ & \;\left.-\overset{N}{\underset{i}{\sum }}\overset{n_{i}}{\underset{j}{\sum }}(1-\delta_{ij})\pi_{ijk}\right]. \end{array}$$

where missing $U_{ij}$ is replaced with $U_{ij}=\frac{1}{k}\sum_{k}P\left[U=1|u_{2i}^{k},t_{ij};\theta^{0}\right]$, and $\pi_{ijk}=\exp\left[-\exp\left[V_{ij}^{T}\gamma +\log\left(u_{2i}^{k}\right)\right]\right]$. Further details about the Mote Carlo sampling techniques and estimation are given in the Supporting Informations. In the M‐step, the parameter γ is obtained through the Newton‐Raphson optimization procedure, where the parameter estimates are iteratively updated conditional on the current estimates of $\omega_{1i}$, $\omega_{2i}$ and $U_{ij}$ given data.

The Newton‐Raphson procedure, applied to $𝔼\left[l_{\beta }\right]$, provides the estimated value of parameters β where γ is estimated from the first part of the likelihood, $\hat{\gamma }$. The expectation of $l_{\beta }$ is simplified as follows.

(10) $$\begin{array}{cc} 𝔼\left[l_{\beta }\right] & =\left[\overset{N}{\underset{i}{\sum }}\overset{n_{i}}{\underset{j}{\sum }}\delta_{ij}\log\left(\lambda_{0}\left(t_{ij}\right)\right)+\overset{N}{\underset{i}{\sum }}\overset{n_{i}}{\underset{j}{\sum }}\delta_{ij}Z_{ij}^{T}\beta \right.\\ & \;-\overset{N}{\underset{i}{\sum }}\overset{n_{i}}{\underset{j}{\sum }}\delta_{ij}\Lambda_{0}\left(t_{ij}\right)\exp\left[Z_{ij}^{T}\beta \right]𝔼\left[u_{1i}|t_{ij}\right]\\ & \;\left.+\overset{N}{\underset{i}{\sum }}\overset{n_{i}}{\underset{j}{\sum }}\delta_{ij}𝔼\left[\log u_{1i}|t_{ij}\right]\right]. \end{array}$$

The third part of the log‐likelihood is the joint frailty model, where the expected likelihood is maximized with respect to α applying the Cauchy approximation [31]. The expected log‐likelihood $l_{\alpha }$ is as follows

$$\begin{array}{ll} l_{\alpha } & =2N\alpha \log\alpha -\alpha N\log2-2N\log\Gamma \left(\alpha \right)\\ & \;-\frac{\alpha }{2}\overset{N}{\underset{i}{\sum }}u_{2i}+\alpha \overset{N}{\underset{i}{\sum }}\log u_{1i}\\ & \;-\left(\alpha -1\right)\overset{N}{\underset{i}{\sum }}\log\left(u_{2i}-2u_{1i}\right). \end{array}$$

where all the expected values on the right‐hand side are evaluated analytically (see Supporting Informations). The standard error is derived from the diagonal of the inverted information matrix using the Louis method [32]. The observed information matrix of $\hat{\gamma }$ using the Louis' method is

(11) $$\begin{array}{cc} I_{\gamma } & =-\frac{1}{K}\overset{K}{\underset{k=1}{\sum }}\frac{\delta^{2}l_{c}}{\delta \gamma \delta \gamma^{T}}+\frac{1}{K}\overset{K}{\underset{k=1}{\sum }}{\left(\frac{\delta l_{c}}{\delta \gamma }\right)}^{T}\left(\frac{\delta l_{c}}{\delta \gamma }\right)\\ & \;-{\left(\frac{1}{K}\overset{K}{\underset{k=1}{\sum }}\frac{\delta l_{c}}{\delta \gamma }\right)}^{T}\left(\frac{1}{K}\overset{K}{\underset{k=1}{\sum }}\frac{\delta l_{c}}{\delta \gamma }\right) \end{array}$$

with the observed information matrix of β and α being

(12) $$I_{\beta }=𝔼\left[-B_{c}\left(\beta \right)\right]-𝔼\left[S_{c}{\left(\beta \right)}^{T}S_{c}\left(\beta \right)\right]-S_{m}{\left(\beta \right)}^{T}S_{m}\left(\beta \right).$$

(13) $$I_{\alpha }=𝔼\left[-B_{c}\left(\alpha \right)\right]-𝔼\left[S_{c}{\left(\alpha \right)}^{T}S_{c}\left(\alpha \right)\right]-S_{m}{\left(\alpha \right)}^{T}S_{m}\left(\alpha \right),$$

where $B_{c}$ is the second derivative of complete‐data likelihood, $S_{c}$ is the gradient vector of complete‐data log‐likelihood and $S_{m}$ is the gradient vector of the observed data log‐likelihood.

#### 2.1.2 Estimation Procedure for Logistic Model

To complete the model formulation, assume $\omega_{1i}=\log\left(u_{1i}\times u_{2i}\right)$ and $\omega_{2i}=\log\left(\frac{u_{2i}}{1-u_{2i}}\right)$, where $u_{1i}∼\Gamma \left(\frac{2}{\alpha },\frac{1}{\alpha }\right)$ and $u_{2i}∼Beta\left(\frac{1}{\alpha },\frac{1}{\alpha }\right)$ and $u_{ij}⊥u_{i^{\prime }j^{\prime }}\forall i\neq i^{\prime }\;\&\;j\neq j^{\prime }$ so that $\omega_{1i}$ is a gamma frailty. The random effect in the cure fraction, $\omega_{2i}$ adds linearly to the respective linear predictor without any range constraints and follows the generalized logistic distribution (type IV) [33]. The joint density of $\omega_{1i}$ and $\omega_{2i}$ has the following functional form:

$$\begin{array}{cc} & f\left(\omega_{1i},\omega_{2i}\right)\\ & \;=\frac{{\frac{1}{\alpha }}^{\frac{2}{\alpha }}\exp\left[-\frac{\exp\left(\omega_{1i}\right)\cdot \left(1+\exp\left(-\omega_{2i}\right)\right)}{\alpha }\right]\exp\left(\frac{2\omega_{1i}}{\alpha }\right)\exp\left(-\frac{\omega_{2i}}{\alpha }\right)}{\Gamma \left(\frac{2}{\alpha }\right)B\left(\frac{1}{\alpha },\frac{1}{\alpha }\right)}. \end{array}$$

Here, $E(e^{\omega_{1i}})=1$, $E(u_{1i}u_{2i})=E(u_{1i})E(u_{2i})=1$ since $u_{1}$ and $u_{2}$ are independent, and $E\left(\omega_{2i}\right)=0$ implies $E(e^{\omega_{2i}})=1$. These are fundamental assumptions for identifiability between the baseline hazard and the frailty term for the model. Moreover, $\omega_{1i}⊥̸\omega_{2i}$ implying $Cor\left(\omega_{1i},\omega_{2,i}\right)=\frac{\psi^{\prime }\left(1/\alpha \right)}{\sqrt{\psi^{\prime }\left(1/\alpha \right)}\sqrt{2\psi^{\prime }\left(1/\alpha \right)}}=0.707$ being the linear correlation coefficient between frailties. Though a fixed correlation is a limitation of the model, it should be noted that the linear correlation is not a good measure of dependence for non‐normal random variables, and it can be empirically verified that the shape of the joint distributions do differ depending on α (see Appendix Figures 7 and 8). Now, conditional on ω, the likelihood function for the observed data is given by

$$L_{c}=\overset{N}{\underset{i=1}{\prod }}\left(\overset{n_{i}}{\underset{j=1}{\prod }}{\left[f\left(t_{ij}|U_{ij}=1,\omega_{1i}\right)\right]}^{\delta_{ij}}{\left[S\left(t_{ij}|\omega_{i}\right)\right]}^{1-\delta_{ij}}\right)f\left(\omega_{i}\right).$$

After applying the log transformation, the conditional likelihood becomes

(14) $$\begin{array}{cc} \log\left(L_{c}\right) & =\overset{N}{\underset{i=1}{\sum }}\overset{n_{i}}{\underset{j=1}{\sum }}\delta_{ij}\log\left[f\left(t_{ij}|U_{ij}=1,\omega_{1i}\right)\right]\\ & \;+\overset{N}{\underset{i=1}{\sum }}\overset{n_{i}}{\underset{j=1}{\sum }}\left(1-\delta_{ij}\right)\log\left[S\left(t_{ij}|\omega_{i}\right)\right]+\overset{N}{\underset{i=1}{\sum }}\log f\left(\omega_{i}\right). \end{array}$$

Let us denote $S\left(t_{ij}|U_{ij}=1,\omega_{1i}\right)=S_{1}$, and $\lambda \left(t_{ij}|U_{ij}=1,\omega_{1i}\right)=\lambda_{1}$ we have from Equation (3)

$$\log\left[f\left(t_{ij}|U_{ij}=1,\omega_{1i}\right)\right]=\log\left(S_{1}\right)+\log\left(\lambda_{1}\right)$$

and

$$\begin{array}{cc} \log\left[S\left(t_{ij}|\omega_{i}\right)\right] & =\log\left(1-\pi_{ij}+\pi_{ij}S_{1}\right)\\ & =\log\left(1-\pi_{ij}\left(1-S_{1}\right)\right)\\ & \approx -\pi_{ij}\left(1-S_{1}\right). \end{array}$$

The corresponding Equation (14)

(15) $$\begin{array}{cc} \log\left(L_{c}\right)= & \overset{N}{\underset{i=1}{\sum }}\overset{n_{i}}{\underset{j=1}{\sum }}\delta_{ij}\left(\log\left(S_{1}\right)+\log\left(\lambda_{1}\right)\right)\\ & +\overset{N}{\underset{i=1}{\sum }}\overset{n_{i}}{\underset{j=1}{\sum }}\left(1-\delta_{ij}\right)\left(-\pi_{ij}\left(1-S_{1}\right)\right). \end{array}$$

The first part of the log‐likelihood Equation (15) only involves the parameter β and $\omega_{1i}$ while the second part contains all parameters, and it is likely to make a very small contribution to the likelihood of β as $\pi_{ij}\leq 1$ and $\left(1-S_{1}\right)\leq 1$. Therefore, both parts of the conditional likelihood function can be used separately to obtain the model parameter estimates, as follows. To estimate β and α we use the following log‐likelihood function

(16) $$\log\left(L_{\beta ,\;\alpha }\right)=\overset{N}{\underset{i=1}{\sum }}\overset{n_{i}}{\underset{j=1}{\sum }}\delta_{ij}\left(\log\left(S_{1}\right)+\log\left(\lambda_{1}\right)\right)+\log\left(f\left(\omega_{2i}|\omega_{1i}\right)\right),$$

where the distribution $f\left(\omega_{2i}|\omega_{1i}\right)$ follows a log‐gamma,

$$f\left(\omega_{2i}|\omega_{1i}\right)=\frac{\exp\left[-\frac{\exp\left(\omega_{1i}-\omega_{2i}\right)}{\alpha }\right]\exp\left[\frac{\omega_{1i}}{\alpha }-\frac{\omega_{2i}}{\alpha }\right]}{\Gamma \left(\frac{1}{\alpha }\right)}.$$

EM algorithm combined with the Newton‐Raphson applied to obtain the estimates, say, $\hat{\beta }$ and $\hat{\alpha }$, where initials are $\beta^{0}$ and $\alpha^{0}$. Then in the second step γ can be estimated from the second part of the likelihood

(17) $$\begin{array}{cc} \log\left(L_{\gamma }\right) & =\overset{N}{\underset{i=1}{\sum }}\overset{n_{i}}{\underset{j=1}{\sum }}\left(1-\delta_{ij}\right)\left[\left(-\pi_{ij}\left(1-S_{1}^{0}\right)\right)\right]\\ & \;+\log\left[f\left(\omega_{1i};\alpha =\alpha^{0}\right)\right], \end{array}$$

where $S_{1}^{0}=S_{1}$ evaluated at $\beta =\hat{\beta }$ and $\alpha =\hat{\alpha }$. Iterate between these two steps until convergence is obtained.

### 2.2 Identical Frailty Model

To make a comparative analysis, another model is designed in such a way that both the recurrence time and cure fraction frailties are identical. The corresponding survival function is given by

$$S\left(t_{ij}|U_{i,j}=1,\omega_{i}\right)=\exp\left[-\Lambda_{0}\left(t_{ij}\right)\exp\left[Z_{ij}^{T}\beta +\omega_{i}\right]\right],$$

where $\Lambda_{0}\left(t_{ij}\right)=\int_{0}^{t_{i,j}}\lambda_{0}\left(t\right)dt$, is the cumulative baseline hazard function, specified as non‐parametric, and the cure status is given by

$$P\left(U_{ij}=1|\omega_{i}\right)=\exp\left[-\exp\left[V_{ij}^{T}\gamma +\omega_{i}\right]\right]=\pi_{ij}\left(U|\omega_{i}\right).$$

The associated density function is given by

$$\begin{array}{cc} f\left(t_{ij}|U_{ij}=1,\omega_{i}\right) & =\exp\left[-\Lambda_{0}\left(t_{ij}\right)\exp\left[Z_{ij}^{T}\beta +\omega_{i}\right]\right]\lambda_{0}\left(t_{ij}\right)\\ & \;\exp\left[Z_{ij}^{T}\beta +\omega_{i}\right].\\ & =S\left(t_{ij}|U_{ij}=1,\omega_{i}\right)\lambda_{ij}\left(t_{ij}|U_{ij}=1,\omega_{i}\right). \end{array}$$

The density function of overall survival is

$$\begin{array}{cc} f\left(t_{ij}|u_{i}\right) & =\lambda_{0}\left(t_{ij}\right)u_{i}\exp\left[Z_{ij}^{T}\beta \right]\\ & \;\times \exp\left[-\Lambda \left(t_{ij}\right)\exp\left[Z_{ij}^{T}\beta \right]u_{i}-\exp\left[V_{ij}^{T}\gamma \right]\right], \end{array}$$

where $\omega_{i}$ followed a gamma distribution with equal shape and scale, α acting as shared frailty for both the recurrence time and cure status components. This frailty accounts for the unobserved heterogeneity, which captures the risk of experiencing the disease or recurrence as well as an individual belonging to the non‐cured group. The idea of using a common gamma frailty for incidence and recurrence processes has been studied in hierarchical cure models [5] to accommodate patient level unobserved heterogeneity. This assumption is plausible in many health data settings where unmeasured subject‐level factors—such as genetic predisposition, immune system functioning, underlying physiology frailty, or multi‐morbid conditions all simultaneously affect the probability of being cured as well as the risk or frequency of future recurrences among the non‐cured. Wienke [34] discussed the plausibility of shared frailties in biomedical recurrences where latent vulnerability is constant for a subject.

#### 2.2.1 Estimation Procedure for Complementary log–log Model in Case of Identical Frailty

To derive the maximum likelihood estimator of the model parameters, the log‐likelihood (Equation 8) is maximized, after setting $\omega_{1i}=\omega_{2i}=\omega_{i}$ and the density of $\omega_{i}$ is replaced by the following univariate form

$$f\left(\omega_{i}\right)=\frac{\alpha^{\alpha }\exp\left[\alpha \omega_{i}\right]\exp\left[-\alpha \exp[\omega_{i}]\right]}{\Gamma \left(\alpha \right)};-\infty <\omega_{i}<\infty .$$

The parameters, γ and β, are estimated using Equations (9) and (10), respectively. The EM algorithm can be performed effectively using Monte Carlo EM where in each iteration, with total K samples of $u_{2}^{1},u_{2}^{2},\ldots ,u_{2}^{K}$ being generated from the density of $f\left(u_{i}|t_{ij}\right)$. The Monte Carlo sample size, K, is 1000 where the whole experiment is repeated 250 times to obtain the highest accuracy. In the M‐step, the parameter γ is obtained through the Newton‐Raphson iterative optimization procedure, with the parameter estimates being updated conditional on the current estimates of $\omega_{i}$ and $U_{ij}$ given data. The second part of the likelihood Equation (8) is associated with Cox proportional hazard and the Newton‐Raphson iterative optimization process is applied to obtain the estimated value of the parameters β using Equation (10) where γ is estimated from Equation (9), $\hat{\gamma }$. The baseline hazard is estimated non‐parametrically.

The α is estimated from the third part of Equation (8) where joint density is replaced by univariate and the likelihood can be expressed as

$$l_{\alpha }\left(\alpha \right)=N\alpha \log\alpha -\alpha \overset{N}{\underset{i}{\sum }}u_{i}+(\alpha -1)\overset{N}{\underset{i}{\sum }}\log u_{i}-N\log\Gamma \left(\alpha \right).$$

The standard error of gamma is derived from Equation (11) by applying the Louis method [32] and the standard error of β and α from the observed information matrix of Equations (12) and (13) respectively.

## 3 Simulation Study

The simulation study of the joint frailty mixture model is conducted to evaluate the performance of the estimators. Two covariates are considered: Z, which is the relevant covariate of the risk of readmission in the long run, and V, which is related to the incidence of the event. This study was conducted considering two scenarios, with a total of N=500 and N=1000 patients, each followed up for 5 years. The covariates Z and V are generated from Bernoulli distributions with a probability of 0.5. For each patient i, the following steps were followed to generate recurrent data.

1. Lost to follow‐up time in days, denoted by $C_{i}$ is generated for each individual from a uniform distribution with a maximum of 5 years.
2. For complementary log–log cure probability, random variable x is generated from a Beta distribution with parameters (α,α), y is generated from gamma distribution (2α,α). The frailty associated with the survival model is defined as $u_{1}=xy$, generated from gamma distribution with parameter (α,α). The frailty connected to disease incidence is $u_{2}=2y$, and also followed a gamma (2α,2α) [30]. In contrast, $u_{1}$ is generated from gamma distribution $\left(\frac{2}{\alpha },\frac{1}{\alpha }\right)$ and $u_{2}$ from Beta with equal shape and scale $\frac{1}{\alpha }$ for logistic cure status.
3. The cure status of the ith individual of the jth recurrent is generated from a Bernoulli distribution. For the complementary log–log model, the probability is $P\left(U_{ij}=1|u_{2i}\right)=\exp\left[-\exp\left[V_{ij}^{T}\gamma \right]u_{2i}\right]$ and $P(U_{ij}=1|\omega_{2i})=\frac{\exp\left[V_{ij}^{T}\gamma +\omega_{2i}\right]}{1+\exp\left[V_{ij}^{T}\gamma +\omega_{2i}\right]}=\pi_{ij}\left(U|\omega_{2i}\right)$ for the logistic model as described in Section 2. If the cure status is 1 (i.e., the patient was not cured), the gap time between successive occurrence, $t_{ij}$ is generated using the survival function $S\left(t_{ij}|\omega_{1i}\right)=\exp\left[-\Lambda_{0}\left(t_{ij}\right)\exp\left[Z_{ij}^{T}\beta \right]u_{1i}\right]$. The Weibull baseline hazard distribution is defined as $f\left(t;\lambda ,\mu \right)=\mu \lambda t^{\mu -1}$ with scale, λ=1 and shape, μ=1.5 for the complementary log–log, and scale, λ=1 and shape, μ=5 for a logistic model, to generate the recurrence gap time. Nevertheless, if the patient is cured, that is, $U_{ij}=0$, and the following recurrence time is censored at $C_{i}$.

**FIGURE 1:**
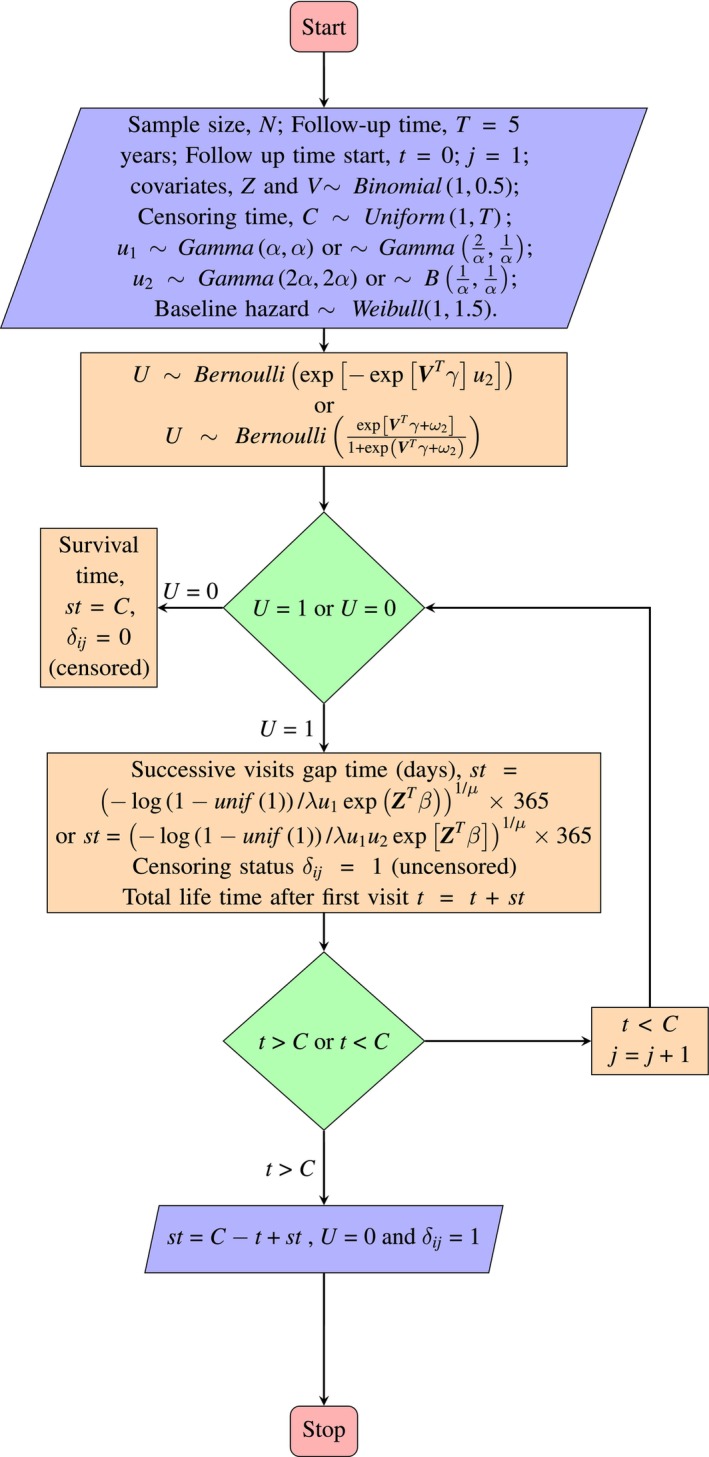
Flowchart of the data generation procedure for each subject in the Monte Carlo simulation.

Iterate Step 3 until $\sum_{i}^{n_{i}}t_{ij}\geq C_{i}$ or $U_{ij}=0$. The Monte Carlo sample size (MCSS) for the E‐step, in the EM procedure, is set to 100, 500, 1000 and 5000. The entire experiment is replicated 250 times, and the validity of the models is tested for different combinations of the values of γ, β and α. The simulation procedure is visually summarized in the flowchart shown in Figure 1, which captures the consequent steps in the analysis.

Table 1 summarizes the average estimated parameters over 250 replications for different MCSSs with a total of 1000 individuals. It includes the average estimated parameters, mean squared error (MSE), and average standard error of complementary log–log model estimated by the Louis [32] method with standard error in parenthesis. The estimates of γ and α are unbiased and consistent across a number of Monte Carlo sample sizes ranging from 100 to 5000. The latent parameter β accounts for the smallest MSE at MCSS 5000, which differs negligibly from the MSE estimated at MCSS = 100. In Table 1, the fifth column shows the mean of the estimated standard error obtained when applying the Louis method, with the empirical standard error of the variance shown in parentheses. The estimated standard error as well as the standard error of the variance, are also close to zero, indicating a small variability which originated in the parameter estimates due to latent variables as compared with observed data. The estimation process of the Monte Carlo EM algorithm took considerably longer time to run with large MCSSs, while similar precision was achieved in a shorter time using smaller MCSSs.

**TABLE 1:** Estimated parameters, SE and MSE from the complementary log–log model for different MC sample sizes and N=1000 based on 250 replication.

Parameters	True parameter	MCSS^a^	Estimate	MSE^b^	ESE^c^(SE^d^)
Incidence $\gamma_{0}$	1	100	1	4.52e‐08	5.27e‐11 (9.09e‐12)
γ	2.5		2.5	5.18e‐08	5.81e‐11 (5.98e‐12)
Latent β	−1		−1	1.36e‐03	0.02 (7.87e‐05)
Frailty α	1.5		1.5	4.20e‐07	0.01 (2.05e‐05)
Cure Rate (SD)			61.78% (6.30)		
Incidence $\gamma_{0}$	1	500	1	4.30e‐08	3.42e‐11 (7.02e‐12)
γ	2.5		2.5	4.90e‐08	5.11e‐11 (9.12e‐12)
Latent β	−1		−1	1.11e‐03	0.02 (7.64e‐05)
Frailty α	1.5		1.5	4.21e‐07	0.01 (2.01e‐05)
Cure Rate (SD)			60.90% (6.44)		
Incidence $\gamma_{0}$	1	1000	1	4.30e‐08	3.42e‐11 (7.02e‐12)
γ	2.5		2.5	4.90e‐08	5.11e‐11 (9.12e‐12)
Latent β	−1		−1	1.72e‐03	0.02 (7.50e‐05)
Frailty α	1.5		1.5	4.21e‐07	0.01 (2.01e‐05)
Cure Rate (SD)			60.90% (6.45)		
Incidence $\gamma_{0}$	1	5000	1	4.47e‐08	3.52e‐11 (7.07e‐12)
γ	2.5		2.5	5.10e‐08	5.25e‐11 (9.33e‐12)
Latent β	−1		−1	4.77e‐04	0.02 (8.22e‐05)
Frailty α	1.5		1.5	5.38e‐06	0.01 (2.26e‐05)
Cure Rate (SD)			61.67% (6.46)		

^a^ MCSS: Monte Carlo sample size.

^b^ MSE: Mean squared error.

^c^ ESE: Estimated empirical standard error.

^d^ SE: Estimated standard error.

Both the complementary log–log and logistic models perform very well under different settings for the parameters, sample sizes and cure rates (Tables 2, 3). The average estimates of γ are unbiased, with a very small standard error of the mean γ for any combination of β and α; even the MSE and SE are close to zero. The small SE reflects the consistency of γ across replications. The SE of the mean estimated β, the MSE, and the estimated SE by the Louis method are also small. The MSE of α tends to be larger, particularly when the true value of α is large, or when β and γ are negative. The issue is mitigated with the large sample sizes. Table 2 presents the estimated parameters for a sample size 1000. The estimates of γ, β and α are unbiased with very low SE and nearly zero MSE.

**TABLE 2:** Estimated parameters, SE and MSE from the complementary log–log cure model for sample size *N* = 1000 based on 250 replication.

Set 1: Baseline hazard with scale 3 and shape 10					Set 2: Baseline hazard with scale 1.5 and shape 3			
Parameter	True parameter	Estimate	MSE^a^	ESE^b^ (SE^c^)	True parameter	Estimate	MSE^a^	ESE^b^ (SE^c^)
**Simulation 1**					**Simulation 5**			
Incidence $\gamma_{0}$	1	0.999	2.34e‐09	2.26e‐07 (1.78e‐08)	1	1	3.26e‐16	1.71e‐06 (1.64e‐07)
γ	3	3.00	2.59e‐09	2.38e‐07 (1.82e‐08)	5	5.00	1.82e‐08	1.73e‐06 (1.65e‐07)
Latent β	3	3.001	3.72e‐08	1.93e‐04 (5.11e‐08)	4	4.00	1.62e‐09	4.03e‐05 (1.96e‐09)
Frailty α	2	2.00	6.32e‐07	5.24e‐04 (3.78e‐07)	1.5	1.499	4.01e‐07	4.91e‐04 (2.91e‐07)
Cure Rate (SD)		90.34% (1.72)			Cure Rate (SD)	86.16% (2.43)		
**Simulation 2**					**Simulation 6**			
Incidence $\gamma_{0}$	1	0.999	5.50e‐14	5.16e‐08 (9.98e‐09)	1	0.999	5.49e‐14	5.16e‐08 (9.99e‐09)
γ	−1	−1.00	9.55e‐08	2.84e‐07 (1.67e‐08)	1.5	−1.5	3.49e‐12	4.09e‐07 (7.12e‐08)
Latent β	0.5	0.500	1.04e‐10	1.02e‐05 (3.11e‐11)	2.5	2.50	2.28e‐12	1.51e‐06 (5.91e‐13)
Frailty α	1.5	1.25	4.33e‐07	4.80e‐06 (6.90e‐12)	1.5	1.03	0.226	6.60e‐06 (9.96e‐12)
Cure Rate (SD)		55.45% (1.69)			Cure Rate (SD)	48.95% (1.78)		
**Simulation 3**					**Simulation 7**			
Incidence $\gamma_{0}$	1	0.999	1.04e‐15	7.51e‐10 (9.74e‐11)	1	0.999	6.84e‐09	7.54e‐08 (5.75e‐09)
γ	−5	−5.00	1.19e‐11	7.97e‐08 (8.73e‐09)	−2	−1.0003	9.55e‐08	2.84e‐07 (1.67e‐08)
Latent β	−4	−3.99	1.35e‐11	3.67e‐06 (2.69e‐12)	−2	0.50	1.04e‐10	1.02e‐05 (3.11e‐11)
Frailty α	0.5	0.50	8.16e‐21	4.04e‐08 (3.23e‐16)	0.75	0.75	2.21e‐15	1.99e‐07 (1.14e‐14)
Cure Rate (SD)		23.09% (0.608)			Cure Rate (SD)	17.88% (1.22)		
**Simulation 4**					**Simulation 8**			
Incidence $\gamma_{0}$	1	0.999	8.49e‐14	8.55e‐08 (8.26e‐09)	1	0.999	6.84e‐09	7.54e‐08 (5.75e‐09)
γ	−1.5	−1.50	2.82e‐12	4.90e‐07 (3.99e‐08)	−1.5	−1.5	9.55e‐08	2.84e‐07 (1.67e‐08)
Latent β	0.75	0.75	1.16e‐11	3.41e‐06 (2.79e‐12)	0.75	0.500	1.04e‐10	1.02e‐05 (3.11e‐11)
Frailty α	1	1.00	3.11e‐07	2.66e‐06 (1.69e‐12)	1	0.75	2.21e‐15	1.99e‐07 (1.14e‐14)
Cure Rate (SD)		46.08% (1.34)			Cure Rate (SD)	17.88% (1.22)		

^a^ MSE: Mean squared error.

^b^ ESE: Estimated empirical standard error.

^c^ SE: Estimated standard error.

**TABLE 3:** Estimated parameters, SE and MSE from the logistic cure model for sample size *N* = 1000 based on 250 replication.

Set 1: Baseline hazard with scale 2 and shape 15					Set 2: Baseline hazard with scale 2 and shape 5			
Parameter	True parameter	Estimate	MSE^a^	ESE^b^ (SE^c^)	True parameter	Estimate	MSE^a^	ESE^b^ (SE^c^)
**Simulation** 1					**Simulation 5**			
Incidence $\gamma_{0}$	1	1	1.37e‐13	6.6e‐08 (3.17e‐08)	1	1	3.26e‐16	1.71e‐06 (1.64e‐07)
γ	1	1	1.46e‐12	8.19e‐08 (3.9e‐08)	1	1	3.44e‐14	2.86e‐08 (6.25e‐09)
Latent β	−0.01	−0.010	7.96e‐17	9.40e‐05 (1.31e‐09)	−0.01	−0.011	8.84e‐18	5.41e‐05 (5.48e‐10)
Frailty α	0.91	0.910	2.62e‐07	0.023 (1.46e‐06)	0.91	0.911	4.01e‐07	0.023 (1.47e‐06)
Cure Rate(SD)		33.25% (0.007)			Cure Rate(SD)	29.45% (0.678)		
**Simulation** 2					**Simulation 6**			
Incidence $\gamma_{0}$	1	0.999	6.98e‐18	5.35e‐10 (1.57e‐10)	1	0.999	5.73e‐19	1.62e‐10 (4.18e‐11)
γ	0.3	0.3	8.87e‐19	9.25e‐10 (2.75e‐10)	0.3	0.3	3.93e‐20	2.95e‐10 (7.74e‐11)
Latent β	0.2	0.20	2.48e‐21	7.00e‐06 (8.94e‐12)	0.2	0.2	2.69e‐22	3.99e‐06 (3.85e‐12)
Frailty α	0.65	0.651	3.95e‐08	0.014 (7.28e‐07)	0.65	0.651	1.44e‐06	0.014 (7.27e‐07)
Cure Rate(SD)		32.61% (0.824)			Cure Rate(SD)	35.77% (0.763)		
**Simulation 3**					**Simulation 7**			
Incidence $\gamma_{0}$	1	0.999	3.90e‐10	4.28e‐11 (1.69e‐11)	1	0.999	3.15e‐20	1.16e‐11 (3.32e‐12)
γ	−0.05	−0.050	2.84e‐12	8.77e‐11 (3.40e‐11)	−0.05	−0.05	1.19e‐21	2.41e‐11 (6.35e‐12)
Latent β	0.2	0.2	2.48e‐21	7.00e‐06 (8.94e‐12)	0.05	0.05	2.98e‐13	1.09e‐06 (2.98e‐13)
Frailty α	0.65	0.65	1.44e‐06	0.014 (7.27e‐07)	0.5	0.51	1.95e‐08	0.011 (1.45e‐06)
Cure Rate(SD)		35.77% (0.762)			Cure Rate(SD)	33.88% (0.858)		
**Simulation 4**					**Simulation 8**			
Incidence $\gamma_{0}$	1	0.999	3.91e‐18	3.91e‐18 (2.43e‐11)	1	0.999	4.35e‐18	1.04e‐09 (2.59e‐10)
γ	−0.5	−0.5	4.58e‐19	4.58e‐19 (5.72e‐11)	0.5	0.51	4.35e‐18	1.04e‐09 (2.59e‐10)
Latent β	0.1	0.1	5.09e‐23	2.64e‐06 (1.58e‐12)	−1	−1	8.91e‐21	9.61e‐06 (1.98e‐11)
Frailty α	0.51	0.51	3.05e‐08	0.013 (2.97e‐06)	0.70	0.7	6.33e‐07	0.016 (6.34e‐07)
Cure Rate(SD)		39.33% (0.939)			Cure Rate(SD)	31.07% (0.739)		

^a^ MSE: Mean squared error.

^b^ ESE: Estimated empirical standard error.

^c^ SE: Estimated standard error.

The complementary log–log model converged across a wide range of cure rates. The first set of true parameter values in Table 2 corresponds to a high cure rate (90.34%). The lowest cure rate, 17.88% was observed in simulation 8. The maximum dependence (0.95) is estimated in simulation 3, where the γ, β are negative and α is small (0.5). As expected, smaller values of α are associated with stronger dependence. In contrast, the lowest correlation was observed in simulation 1, which exhibited a very high cure rate outcome which is practically reasonable. Overall, the results indicate that the frailty parameter α is inversely related to the degree of dependence or homogeneity. The logistic model is more restrictive than the complementary log–log model, as it can handle cure rates only up to approximately 40% with the lowest convergent value observed at 29%. Therefore, further methodological investigation is required to relax the boundary constraints of cure rates. Consequently, the estimators are consistent, as they converge to the true parameter value as the sample size increases. The performance of prediction of survival model is obtained by using the Concordance Index (C‐index) [35]. The simulated data set was partitioned into train data and rest data with a 70:30 ratio, and the very small gap between the C‐indexes of train and test indicates no overfitting of the model.

Therefore, both proposed models can adequately handle different cure rates, as well as individual‐level heterogeneity in disease exposure (non‐cured) and, in the long‐run, risk of hospital readmission.

## 4 Analysis of Colorectal Cancer Hospital Re‐Admission Data

Successive rehospitalization of patients diagnosed with colorectal cancer from a prospective cohort study published by González [25], available in the frailtypack [36] package in R, was used in this study to explore the proposed models. Among 523 patients identified with colorectal cancer between January 1996 and December 1998, 403 underwent surgery. These 403 patients were followed until 2002, and their age, sex, chemotherapy, Dukes stage, and tumor site were recorded as covariates.

The binary variable chemo presents whether the patient received chemotherapy or not. Sex is classified as male or female. Dukes represents the tumoral stages and is coded as 1 for A‐B; 2 for C, and 3 for D. The Charlson is the Comorbidity Charlson index coded as 0 for Index 0, 1 for Index 1‐2, and 3 for Index ≥ 3. The data provide the gap times (in days) of successive hospitalizations after the date of surgery. The primary outcome measure is the time to hospital readmission related to colorectal cancer following tumor‐removal surgery.

The date of surgery was taken as the beginning of the study period. The first readmission time was considered as the time between date of the surgical procedure and the first readmission to the hospital due to the colorectal cancer. The following readmission times were considered as the difference between last discharge and the current hospital admission date. In total 861 readmissions were recorded due to recurrence of colorectal cancer.

About 50% of the patients had no recurrence during the entire 5 years. Patients who survived 5 years had a higher chance of being cured, and early‐stage detection of colorectal cancer is associated with higher survival and cure rates. According to the National Cancer Institute, colon cancer is highly treatable and often a curable disease after surgery, especially when it is localized to the bowel [37]. A recent study found around 70.3% of colorectal cancer patients survived and were recurrence‐free, while 8.4% were alive after recurrence [38].

**FIGURE 2:**
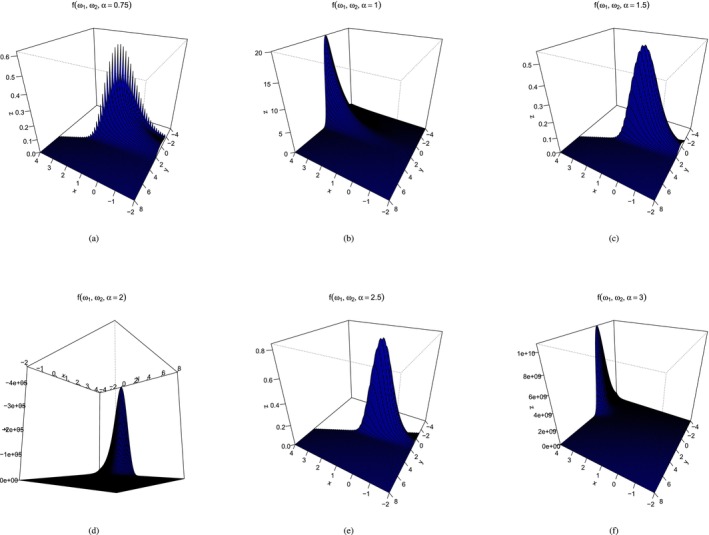
Dependence of frailties of complementary log–log model for different α. (a), (b), (c), (d), (e), (f).

Empirically, readmission data consists of a fraction of long‐term survivors indicating the presence of cured patients who are no longer vulnerable to disease‐related death. The K‐M survival curves of the gap times for the first four successive visits are presented in Figure 2. The survival curve for time to first readmission differs substantially from the other curves as well as from the time‐to‐death curve. The recurrence‐free rate is higher compared to the subsequent visits, and the tail of the death curve is above 0.8. The data indicates the presence of long‐term survivors as the right tails of both the first and fourth visit curves drop to zero, suggesting that the longest times are uncensored. In contrast, the second and the third readmission curves, as well as the final recurrence before death, remain flat toward the end of the follow‐up and indicate that no further events were observed during this period. The time‐to‐death curve also remains steady, indicating there are some reasons other than the event, existing for censoring.

The K‐M survival curves of the gap times for the first four successive visits classified by tumor stage are presented in Figure 3. The recurrence‐free rate is higher across all tumor stages for the first visits compared to subsequent three visits. In addition, the survival curve for the time of the first visit at tumor stage A‐B is significantly different from those at stages C, and D, with the longest times being uncensored. The K‐M curves of the 1st, 2nd, 3rd and 4th readmissions, stratified by other covariates, are given in Figures 4–6.

For an individual patient, hospital readmission and death are correlated resulting in dependent censoring. This dependence may arise due to patient‐specific unobserved random effects, for example, inflammatory bowel disease or cancer stage [3]. Therefore, the proposed mixture cure frailty models, which account for dependent censoring sourced from cure status, may be appropriate for modeling successive recurrent event data with a cure fraction and a dependent terminal event.

Table 4 presents the summary results estimated from the mixture cure frailty models. All negative coefficients correspond to a decrease of the hazard of being non‐cured individuals, indicating low recurrence probability as well as longer survival. In the cure component (clog–log link), the coefficient for female is positive and greater than 1 indicating that females have an approximately 11% lower risk of being susceptible to recurrence compared to males (the reference category). Equivalently, females have a higher probability of being cured compared with males. In the survival component, the regression coefficient for female is $\beta_{\text{female}}=-1.639,$ which corresponds to hazard ratio of exp(−1.639)≈0.19. This implies, among non‐cured individuals, that females have about an 81% lower hazard than males for readmission. This dual protective effect highlights a strong gender‐related difference in both the incidence and progression of the event. Chemotherapy has been found significantly associated with hospital readmission; in the cure component (clog–log link), the coefficient for receiving chemotherapy is positive and shows a lower probability of 11% of being non‐cured compared with patients who did not receive chemo. In the survival component, among non‐cured patients, individuals receiving chemotherapy have a 93% lower hazard than those who did not receive chemotherapy. In the long run, readmission is more likely to occur for non‐cured male patients and those who did not receive any treatment. Gender difference was reported previously in this data [25] with respect to rehospitalization [5] and in both incidence and rehospitalization [3].

**TABLE 4:** Results of the survival model for successive rehospitalization of patients diagnosed with colorectal cancer.

Effects	Estimates^a^	Estimates^b^	Estimates^c^
**Incidence (cure status) component**			
Covariates	Complementary log–log	Logistic component	Identical Frailty Model
Intercept	1.47	0.577	2.72
Gender			
Male (Ref)			
Female	1.11 (2.0e‐03)	1.37 (2.24e‐01)	2.72 (1.7e‐06)
Chemo			
NonTreated (Ref)			
Treated	1.11 (2.0e‐03)	1.50 (2.33e‐01)	2.72 (1.8e‐06)
Dukes			
A‐B (Ref)			
C	1.14 (2.0e‐03)	1.22 (2.67e‐01)	2.72 (2.1e‐06)
D	1.13 (3.0e‐03)	1.16 (3.20e‐01)	2.72 (2.6e‐06)
Charlson index			
0 (Ref)			
1‐2	1.12 (4.0e‐03)	1.89 (1.67e‐01)	2.72 (2.1e‐06)
≥3	1.10 (2.0e‐03)	1.26 (4.85e‐01)	2.72 (2.1e‐06)
**Cox proportional hazard component**			
Gender			
Male (Ref)			
Female	0.194 (2.80e‐02)	1.65 (5.55e‐03)	0.173 (1.8e‐02)
Chemo			
NonTreated (Ref)			
Treated	0.037 (6.0e‐03)	3.29 (7.70e‐03)	0.059 (1.4e‐02)
Dukes			
A‐B (Ref)			
C	0.035 (1.8e‐02)	11.71 (8.71e‐03)	0.060 (1.7e‐02)
D	0.056 (2.6e‐02)	14.20 (1.21e‐02)	0.088 (2.5e‐02)
Charlson index			
0 (Ref)			
1‐2	0.150 (4.4e‐02)	3.98 (2.05e‐02)	0.238 (1.4e‐02)
≥3	0.221 (2.5e‐02)	1.87 (1.15e‐02)	0.302 (2.3e‐02)
α	0.954 (4.150e‐02)	0.10 (6.04e‐04)	0.099 (5.2e‐04)
AIC	13083.64	79503.05	8350.12

^a^ Correlated frailty complementary log–log incidence and Cox‐proportional hazard model.

^b^ Correlated frailty logistic incidence and Cox‐proportional hazard model.

^c^ Identical frailty complementary log–log incidence and Cox‐proportional hazard model.

^d^ SE: Standard error.

The relative risk of recurrence is found to be similar across the respective categories of both advanced Dukes stages and high Charlson comorbidity index, which is consistent with findings from previous research [39]. The incidence risk of being non‐cured is approximately 14% lower at tumor stages C and D compared to stage A‐B. Meanwhile, non‐cured patients at stage C or D have a lower risk of recurrence compared to those at stage A‐B. A patient with a Charlson comorbidity index of 1−2 has a 0.109 times lower risk of being non‐cured, while those with an index ≥3 have a 0.085 times lower risk compared to a patient with a comorbidity index 0. In addition, the risk of recurrence for non‐cured patients is 73% and 59% lower, respectively, compared to those with an index of 0. In terms of incidence risk, Dukes stage C and D, and as well as higher comorbidity, decrease the likelihood of being cured in both models which is also consistent with previous research [3]. In the incidence component of the logistic model, females have 1.37 times higher odds of being non‐cured compared with males, and patients who received chemotherapy have 1.50 times higher odds of being non‐cured compared with those who did not receive chemotherapy, after accounting for subject‐specific random effects. Therefore, female patients are associated with a 37% increase in the odds of being non‐cured, while receipt of chemotherapy is associated with a 50% increase in the odds of being non‐cured, conditional on the subject‐specific random effect. Moreover, among non‐cure patients, female patients are 1.65% more likely and patients who received chemotherapy are 3.29% more likely to relapse. Thus, a significant difference is observed between the male and female patients regarding the probability of both being cured and the risk of recurrence. The odds of being non‐cured for tumor stages C and D are nearly comparable. Similar patterns are observed for the coefficients associated with the Charlson comorbidity index. Among non‐cured patients, those diagnosed at tumor stages C and D have 11.71% and 14.20% higher odds of relapse, respectively, compared with patients at stage A−B. Similarly, patients with a Charlson comorbidity index of 1−2 and ≥3 have 3.98% and 1.87% higher odds of relapse, respectively, compared with those with an index of 0. However, in the identical frailty model (column 4), the incidence estimates indicate an equal hazard ratio (HR=2.72) for being non‐cured across all covariates and categories. As a result, the estimated probability of cure is 1−exp(−exp(1.00))=1−exp(−2.718)=0.934. Therefore, the model predicts a cure probability of 0.934 which is very high close to 1 for the baseline category that is, the male patients who did not receive chemotherapy, had tumoral stage A‐B, and a Charlson comorbidity index 0.

The parameter α controls the degrees of unobserved heterogeneity. The estimated frailty parameter, α, from an identical frailty model is 0.099 with a standard error of 5.2e−04 which is lower than the estimated frailty parameter of 0.954 with standard error 1.0e−03 (see Table 4). This suggests that heterogeneity in the hazard rate for hospital readmission is smaller under the identical frailty assumption compared to the dependent frailty complementary log–log model. However, α estimated from logistic cure incidence models is 0.01 with very small standard error. Therefore, the dependent frailty model captures greater heterogeneity than the identical frailty model.

The exponentiated 99% confidence interval of hazard ratios indicates (calculated but not shown here) statistical significance, as all intervals exclude 1. The narrow range of the confidence interval was obtained because of small standard errors in the estimated parameters. The Akaike Information Criterion (AIC), estimated from the observed likelihood of the mixture complementary log–log cure frailty model is smaller compared to the logistic cure frailty model.

The algorithm used for parameter estimation is very flexible, allowing γ to converge on average after 65 iterations, β after 55 iterations, and α after 48 iterations. The entire estimation process of hospital readmission for colorectal cancer data, using a MCSS of 1000 and a tolerance level of 0.001, takes approximately 40 s to complete.

## 5 Discussion

This study has developed a semi‐parametric mixture cure model with dependent frailty for a cluster of structured recurrent event data. This technique is more advantageous than existing frailty mixture cure models found in the literature with respect to the provision for correlated and non‐Gaussian random effects between cure and recurrence components. The possibility of cure is updated after each recurrence rather than treating the patient as non‐cured forever after the first visit. The separate but subject specific correlated frailties for both incidence and latent components were adopted with a view to overcoming the limitation of existing random effects models requiring shared (identical) or proportional random effects.

A likelihood‐based estimation approach is formulated where Monte Carlo EM is used to estimate the unknown parameters as the integral in the E‐step could not be solved analytically. The computational competency of the proposed complementary log–log model provides less bias and prompt convergence to the true value. The model is validated through simulation, as well as applying hospital readmission data on colorectal cancer patients with a cure rate of around 47%. The MSE estimated from large samples is close to zero, and the small estimated standard deviation indicates the good fit of the model. The MSEs of the regression parameters estimated from the simulated data are very small and even became negligible (i.e., close to zero) with large sample sizes. Therefore, these findings suggest that the model closely approximates the true underlying process, minimizing systematic errors. This model is substantiated for any cure rate as well as different follow‐up times and can handle the correlated heterogeneity in disease incidence together with long term survival. The negligible bias, small SE, and low MSEs remain stable across various combinations of true parameters, allowing for confident inference about the true value of the model. The method of ensuring consistency of the results in combinations of parameters suggests that it can be generalized effectively, making it a reliable choice in diverse scenarios.

The second proposed model allows logistic cure status in the mixture frailty model. To mitigate computational challenges, the EM algorithm is applied to the observed data likelihood. The marginal densities of the recurrent times, as well as the conditional expectations which are necessary in EM algorithm, are derived using the hyper‐geometric function as direct integration is analytically intractable. Both the simulated and observed hospital readmission data used to validate the proposed model yield accurate estimates within reasonable error bounds, indicating the models adequately capture the data‐generating process in both cases. The logistic model is relatively simple and results in a less time‐consuming estimation process, although it needs further investigation to valid its performance across a wide range of cure rates. Moreover, in this model set up, the correlation of frailties is assumed fixed (correlation 0.707). Given this constraint, the logistic mixed model is less flexible than the complementary log–log mixture cure model, which in turn exhibits superior efficiency and may consequently be regarded as a suitable approach for modeling the underlying recurrent event process. A further extension of the logistic model with separate parameters governing two frailties, instead of one parameter (α), may be tried and, thus, could be the subject for future research.

In the simulation studies, the baseline hazard was modeled as a Weibull distribution, which is also appropriate for different combinations of shape and scale. Correlated frailties were modeled as bivariate gamma where marginals are gamma with a mean value is 1. The proposed joint frailty mixture cure models, which currently address right censoring, could be further extended to accommodate interval censoring. Another potential extension involves using a copula function to model the connection of the random effects of exposure on both the cured probability and the hazard of failure for the non‐cure to relax the strong distributional assumptions about frailty distribution.

## Author Contributions

**Nasrin Sultana:** model derivation, analysis, writing manuscript. **Moudud Alam:** conceptualization, supervision, review, and editing of the manuscript. **Md Hasinur Rahaman Khan:** conceptualization (supporting), supervision, review, and editing of the manuscript.

## Funding

This research was supported by the Bangabandhu Science and Technology Fellowship Trust under the Ministry of Science and Technology, Bangladesh.

## Conflicts of Interest

The authors declare no conflicts of interest.

## Supporting information


**Data S1**: Supporting Information.

## Data Availability

The data that support the findings of this study are openly available in The Comprehensive R Archive Network at DOI: https://10.32614/CRAN.package.frailtypack.

## Appendix A

### Densities and Expectations of Complementary log–log Cure Model

#### Joint Conditional Moments of $u_{1i},u_{2i}$

$$\begin{array}{ll} & f\left(u_{1i},u_{2i}|t_{ij}\right)=\frac{1}{(\alpha +1)\;\Gamma {\left(\alpha \right)}^{2}}{\left(\exp[{V_{\mathrm{ij}}}^{T}\gamma ]+\frac{\alpha }{2}\right)}^{\alpha }\\ & \;{\left(\alpha +2\exp[{V_{\mathrm{ij}}}^{T}\gamma ]+\Lambda_{0}(t_{ij})\exp[Z_{ij}^{T}\beta ]\right)}^{\alpha +1}\\ & \times \exp\left[-u_{2i}\left(\exp\left[{V_{\mathrm{ij}}}^{T}\gamma \right]+\frac{\alpha }{2}\right)-u_{1i}\Lambda_{0}(t_{ij})\exp\left[{Z_{\mathrm{ij}}}^{T}\beta \right]\right]\\ & \;u_{1i}^{\alpha }\;{\left(u_{2i}-2u_{1i}\right)}^{\alpha -1}. \end{array}$$

Let us consider the constant $a=\exp\left[{V_{\mathrm{ij}}}^{T}\gamma \right]+\frac{\alpha }{2},\;b=\Lambda_{0}\left(t_{ij}\right)\exp\left[Z_{ij}^{T}\beta \right]$, and $\;c=\alpha +2\exp\left[{V_{\mathrm{ij}}}^{T}\gamma \right]+b.$ The joint moment generating function of $\left(u_{1i},u_{2i}\right)$ conditional on $t_{ij}$ is with support $u_{1i}>0$, $u_{2i}>2u_{1i}$ obtained

$$M_{(u_{1},u_{2})|t}(s_{1},s_{2})=\frac{\alpha }{\alpha +1}\frac{a^{\alpha }c^{\alpha +1}}{{\left(a-s_{2}\right)}^{\alpha }{\left(b+2(a-s_{2})-s_{1}\right)}^{\alpha +1}}.$$

Therefore,

$$\begin{array}{ll} 𝔼\left[u_{1i}|t_{ij}\right] & =\frac{\alpha }{\left[\alpha +2\exp\left[V_{ij}^{T}\gamma \right]+\Lambda_{0}\left(t_{ij}\right)\exp\left[Z_{ij}^{T}\beta \right]\right]},\\ 𝔼\left(u_{2}|t_{ij}\right) & =\frac{\alpha }{\left(\exp\left[V_{ij}^{T}\gamma \right]+\frac{\alpha }{2}\right)}\\ & \;+\frac{2(\alpha +1)}{\left(\Lambda_{0}\left(t_{ij}\right)\exp\left[Z_{ij}^{T}\beta \right]+2\exp\left[V_{ij}^{T}\gamma \right]+\alpha \right)},\\ \mathrm{Var}\left(u_{1}|t_{ij}\right) & =\frac{\alpha }{{\left(\Lambda_{0}\left(t_{ij}\right)\exp\left[Z_{ij}^{T}\beta \right]+2\exp\left[V_{ij}^{T}\gamma \right]+\alpha \right)}^{2}},\\ \mathrm{Var}(u_{2}|t_{ij}) & =\frac{\alpha }{{\left(\exp\left[V_{ij}^{T}\gamma \right]+\frac{\alpha }{2}\right)}^{2}}\\ & \;+\frac{4(\alpha +1)}{{\left(\Lambda_{0}\left(t_{ij}\right)\exp\left[Z_{ij}^{T}\beta \right]+2\exp\left[V_{ij}^{T}\gamma \right]+\alpha \right)}^{2}},\\ \mathrm{Cov}\left(u_{1},u_{2}|t_{ij}\right) & =\frac{2(\alpha +1)}{{\left(\Lambda_{0}\left(t_{ij}\right)\exp\left[Z_{ij}^{T}\beta \right]+2\exp\left[V_{ij}^{T}\gamma \right]+\alpha \right)}^{2}}. \end{array}$$

Substituting back a and b gives the expression in covariates and baseline hazard.

#### Marginal Density of Recurrent Events

$$\begin{array}{ll} & f\left(t_{ij}\right)=\frac{\left(\alpha +1\right)\alpha^{2\alpha }2^{-\alpha }\lambda_{0}\left(t_{ij}\right)\exp\left[Z_{ij}^{T}\beta \right]}{{\left[\exp\left[V_{ij}^{T}\gamma \right]+\frac{\alpha }{2}\right]}^{\alpha }{\left[\alpha +2\exp\left[V_{ij}^{T}\gamma \right]+\Lambda_{0}\left(t_{ij}\right)\exp\left[Z_{ij}^{T}\beta \right]\right]}^{\alpha +1}}. \end{array}$$

#### Density and Expectation of Frailty Associated With Complementary log–log Component Given Frailty Associated With Hazard Model and Each Time

Joint density of $f\left(u_{1i},u_{2j},t_{ij}\right)$ is obtained by $f\left(u_{1i},u_{2j},t_{ij}\right)=f\left(t_{ij}|u_{1i},u_{2j}\right)f\left(u_{1i},u_{2i}\right)$ which is given by

$$\begin{array}{ll} & f\left(u_{1i},u_{2j},t_{ij}\right)=\frac{2^{-\alpha }\alpha^{2\alpha }\lambda_{0}\left(t_{ij}\right)\exp\left[Z_{ij}^{T}\beta \right]}{\Gamma {\left(\alpha \right)}^{2}}\\ & \;\exp\left[-u_{2i}\exp\left[V_{ij}^{T}\gamma \right]-u_{1i}\Lambda_{0}\left(t_{ij}\right)\exp\left[Z_{ij}^{T}\beta \right]\right]\\ & \;\exp\left[-\frac{\alpha u_{2i}}{2}\right]{u_{1}}^{\alpha }{\left[u_{2i}-2u_{1i}\right]}^{\alpha -1}. \end{array}$$

Marginal density of $f\left(u_{2i}|u_{1i},t_{ij}\right)=\frac{f\left(u_{1i},u_{2j},t_{ij}\right)}{f\left(u_{1i},t_{ij}\right)}$ is given by

$$\begin{array}{ll} f\left(u_{2i}|u_{1i},t_{ij}\right) & =\frac{{\left[\exp V_{ij}^{T}\gamma +\frac{\alpha }{2}\right]}^{\alpha }}{\Gamma \left(\alpha \right)}{\left(u_{2i}-2u_{1i}\right)}^{\alpha -1}\\ & \;\exp\left[-u_{2i}\left(\exp\left[V_{ij}^{T}\gamma \right]+\frac{\alpha }{2}\right)\right]\\ & \;\exp\left[u_{1i}\left(2\exp\left[V_{ij}^{T}\gamma \right]+\alpha \right)\right]. \end{array}$$

#### Expectation of Being Cure Given Data

$$\begin{array}{ll} & 𝔼\left[U|t_{ij}>t\right]=\frac{P\left(U_{ij}=1\right)S\left(t_{ij}|U_{ij}=1\right)}{1-P\left(U_{ij}=1\right)+P\left(U_{ij}=1\right)S\left(t_{ij}|U_{ij}=1\right)}.\\ & \;=\frac{{\left[1+\frac{\Lambda_{0}\left(t_{ij}\right)\exp\left[Z_{ij}^{T}\beta \right]}{\alpha }\right]}^{-\alpha }}{{\left[1+\frac{\exp\left[V_{ij}^{T}\gamma \right]}{2\alpha }\right]}^{-2\alpha }+{\left[1+\frac{\Lambda_{0}\left(t_{ij}\right)\exp\left[Z_{ij}^{T}\beta \right]}{\alpha }\right]}^{-\alpha }-1},\\ & \text{where}\;P\left[U=1|u_{2i},t_{ij}\right]\\ & \;=\frac{{\left[2\alpha \right]}^{2\alpha }\exp\left[-u_{2i}\left[\exp\left[V_{ij}^{T}\gamma \right]+2\alpha \right]\right]u_{2i}^{2\alpha -1}}{\Gamma 2\alpha }. \end{array}$$

#### Necessary Expectations to Logarithm Components to Implement EM Algorithm

$$\begin{array}{ll} & 𝔼\left[\log u_{1i}|t_{ij}\right]=\psi \left(\alpha +1\right)-\ln\left[\alpha +2\exp\left(V_{ij}^{T}\gamma \right)\right.\\ & \;+\left.\Lambda_{0}\left(t_{ij}\right)\exp\left(Z_{ij}^{T}\beta \right)\right]. \end{array}$$

$$\begin{array}{ll} & 𝔼\left[\log u_{1i}|t_{i.}\right]=\psi \left(\alpha +1\right)-\ln\left[\alpha +2\underset{j}{\sum }\exp\left(V_{ij}^{T}\beta \right)\right.\\ & \;+\left.\underset{j}{\sum }\Lambda_{0}\left(t_{ij}\right)\exp\left(Z_{ij}^{T}\beta \right)\right]. \end{array}$$

$$\begin{array}{ll} & 𝔼\left[u_{2i}-2u_{1i}|t_{ij}\right]=\psi \left(\alpha \right)-\ln\left[\exp\left(V_{ij}^{T}\gamma \right)+\frac{\alpha }{2}\right],\\ & \;\;\text{where}\;\psi_{u_{2i}-2u_{1i}}\left(\alpha \right)=\frac{d\ln\Gamma \left(\alpha \right)}{x}. \end{array}$$

$$\begin{array}{l} \text{Therefore,}\;𝔼\left[u_{2i}-2u_{1i}|t_{i.}\right]=\psi \left(\alpha \right)-\ln\left[\underset{j}{\sum }\exp\left(V_{ij}^{T}\gamma \right)+\frac{\alpha }{2}\right]. \end{array}$$

$$\begin{array}{ll} 𝔼\left[\log u_{2i}|t_{i.}\right] & \approx \log 𝔼\left[u_{2i}|t_{i.}\right]-\frac{V\left[u_{2i}|t_{i.}\right]}{2𝔼\left[u_{2i}^{2}|t_{i.}\right]}\\ & =\log 𝔼\left[u_{2i}|t_{i.}\right]-\frac{1}{2}+\frac{𝔼{\left[u_{2i}|t_{i.}\right]}^{2}}{2𝔼\left[u_{2i}^{2}|t_{i.}\right]}. \end{array}$$

$$\begin{array}{ll} & 𝔼\left[u_{2i}^{2}|t_{i.}\right]=\frac{\left(\alpha +2\right)}{\left(\alpha +1\right)\prod_{j}^{n_{i}}{\left[\frac{\alpha }{2}+\exp\left[V_{ij}^{T}\gamma \right]\right]}^{2}}\\ & \;+\frac{4\left(\alpha +1\right)\left(\alpha +2\right)}{\begin{array}{l} \prod_{j}^{n_{i}}\left[\frac{\alpha }{2}+\exp\left[V_{ij}^{T}\gamma \right]\right]\left[\alpha +2\sum_{j}^{n_{i}}\exp\left[V_{ij}^{T}\gamma \right]\right.\\ \left.+\sum_{j}^{n_{i}}\Lambda_{0}\left(t_{ij}\right)\exp\left[Z_{ij}^{T}\beta \right]\right] \end{array}}\\ & \;+\frac{4\left(\alpha +2\right)\left(\alpha +3\right)}{{\left[\alpha +2\sum_{j}^{n_{i}}\exp\left[V_{ij}^{T}\gamma \right]+\sum_{j}^{n_{i}}\Lambda_{0}\left(t_{ij}\right)\exp\left[Z_{ij}^{T}\beta \right]\right]}^{2}}. \end{array}$$

$$\begin{array}{ll} & 𝔼\left[\exp\left[-\Lambda_{0}\left(t_{ij}\right)u_{1i}\exp\left(Z_{ij}^{T}\beta \right)\cdot \exp\left[-\exp\left[V_{ij}^{T}\gamma \right]u_{2i}\right)\right)\right]\\ & \;=\frac{\alpha^{\alpha }{\left[\alpha +2\sum_{j}^{n_{i}}\exp\left[V_{ij}^{T}\gamma \right]+\sum_{j}^{n_{i}}\Lambda_{0}\left(t_{ij}\right)\exp\left[Z_{ij}^{T}\beta \right]\right]}^{\alpha +1}}{\begin{array}{l} \prod_{j}^{n_{i}}{\left[\frac{\alpha }{2}+2\exp\left[V_{ij}^{T}\beta \right]\right]}^{\alpha }\left[\alpha +4\sum_{j}^{n_{i}}\exp\left[V_{ij}^{T}\gamma \right]\right.\\ {\left.+2\sum_{j}^{n_{i}}\Lambda_{0}\left(t_{ij}\right)\exp\left[Z_{ij}^{T}\beta \right]\right]}^{\alpha } \end{array}}. \end{array}$$

### Densities and Expectations of Logistic Cure Model

#### Joint Density of Frailties and t

The joint density of recurrent time and frailties can be found in the flowing form

$$\begin{array}{ll} & f\left(t_{ij},\omega_{1i},\omega_{2i}\right)=\frac{\begin{array}{l} {\left(\frac{1}{\alpha }\right)}^{\frac{2}{\alpha }}\lambda_{0}(t_{ij})\exp\left[V_{ij}^{T}\gamma +Z_{ij}^{T}\beta \right]\\ \exp\left[\omega_{1i}+\frac{2\omega_{1i}}{\alpha }\right]\exp\left[\omega_{2i}\left(1-\frac{1}{\alpha }\right)\right] \end{array}}{\Gamma \left(\frac{2}{\alpha }\right)\;B\left(\frac{1}{\alpha },\frac{1}{\alpha }\right)}\\ & \;\times \frac{\begin{array}{l} \exp\left[-\Lambda_{0}(t_{ij})\exp\left(Z_{ij}^{T}\beta +\omega_{1i}\right)\right.\\ -\;\left.\frac{1}{\alpha }\left(\exp(\omega_{1i})+\exp(\omega_{1i}-\omega_{2i})\right)\right] \end{array}}{1+\exp\left[V_{ij}^{T}\gamma +\omega_{2i}\right]}. \end{array}$$

#### Joint Density of t and $\omega_{2i}$

$$\begin{array}{ll} & f(\omega_{2i},t_{ij})=\frac{\begin{array}{l} {\left(\frac{1}{\alpha }\right)}^{\frac{2}{\alpha }}\lambda_{0}(t_{ij})\exp\left[V_{ij}^{T}\gamma +Z_{ij}^{T}\beta \right]\\ \exp\left[\omega_{2i}\left(1-\frac{1}{\alpha }\right)\right]\Gamma \left(1+\frac{2}{\alpha }\right) \end{array}}{\begin{array}{l} \Gamma \left(\frac{2}{\alpha }\right)\;B\left(\frac{1}{\alpha },\frac{1}{\alpha }\right)\;\left(1+\exp\left[V_{ij}^{T}\gamma +\omega_{2i}\right]\right)\\ {\left[\Lambda_{0}(t_{ij})\exp\left[Z_{ij}^{T}\beta \right]+\frac{1}{\alpha }+\frac{1}{\alpha }\exp(-\omega_{2i})\right]}^{1+2\alpha } \end{array}}. \end{array}$$

#### Marginal Density of t

Now comparing Equations (13) and (14) we see that the marginal density of t is obtained (up to some proportionality constant) by

$$\begin{array}{l} f(t_{ij})\propto \frac{\exp\left[\omega_{2i}\left(1-\frac{1}{\alpha }\right)\right]}{\begin{array}{l} {\left[\Lambda_{0}\left(t_{ij}\right)\exp\left(Z_{ij}^{T}\beta \right)+\frac{1}{\alpha }+\frac{1}{\alpha }\exp\left(-\omega_{2i}\right)\right]}^{1+\frac{2}{\alpha }}\\ \left[1+\exp(V_{ij}^{T}\gamma +\omega_{2i})\right] \end{array}}. \end{array}$$

where the proportionality constant involves all the terms in $f\left(\omega_{2i},t_{ij}\right)$ which do not involve $\omega_{2i}$. Now to make the above a probability density function we have to find

$$\begin{array}{l} \Psi =\int_{-\infty }^{\infty }\frac{\exp\left[\omega_{2i}\left(1-\frac{1}{\alpha }\right)\right]}{\begin{array}{l} {\left[\Lambda_{0}\left(t_{ij}\right)\exp(Z_{ij}^{T}\beta )+\frac{1}{\alpha }+\frac{1}{\alpha }\exp(-\omega_{2i})\right]}^{1+\frac{2}{\alpha }}\\ \left[1+\exp\left[V_{ij}^{T}\gamma +\omega_{2i}\right]\right] \end{array}}d\omega_{2i}. \end{array}$$

Let $\exp\left[\omega_{2i}\right]=m$, so that $d\omega_{2i}=\frac{dm}{m}$. Then the Equation is

$$\begin{array}{ll} \Psi & =\int_{0}^{\infty }\frac{m^{\left(1-\frac{1}{\alpha }\right)}}{\begin{array}{l} {\left[\Lambda_{0}\left(t_{ij}\right)\exp\left[Z_{ij}^{T}\beta \right]+\frac{1}{\alpha }+\frac{1}{\alpha m}\right]}^{1+\frac{2}{\alpha }}\\ \left[1+m\exp\left[V_{ij}^{T}\gamma \right]\right] \end{array}}\frac{dm}{m}, \end{array}$$

or,

$$\begin{array}{ll} \Psi & =\int_{0}^{\infty }\frac{m^{\left(-\frac{1}{\alpha }\right)}m^{\left(1+\frac{2}{\alpha }\right)}\alpha^{\left(1+\frac{2}{\alpha }\right)}}{\begin{array}{l} {\left[1+m+m\alpha \Lambda_{0}\left(t_{ij}\right)\exp\left[Z_{ij}^{T}\beta \right]\right]}^{1+\frac{2}{\alpha }}\\ \left[1+m\exp\left[V_{ij}^{T}\gamma \right]\right] \end{array}}dm \end{array}$$

or,

$$\begin{array}{l} \Psi =\int_{0}^{\infty }\frac{m^{\left(1+\frac{1}{\alpha }\right)}\alpha^{\left(1+\frac{2}{\alpha }\right)}}{\begin{array}{l} {\left[1+m+m\alpha \Lambda_{0}\left(t_{ij}\right)\exp\left[Z_{ij}^{T}\beta \right]\right]}^{1+\frac{2}{\alpha }}\\ \times \left[1+m\exp\left[\gamma^{T}V_{ij}\right]\right] \end{array}}dm, \end{array}$$

or,

$$\begin{array}{ll} \Psi & =\alpha^{\left(1+\frac{2}{\alpha }\right)}\int_{0}^{\infty }\left(\frac{m^{\left(1+\frac{1}{\alpha }\right)}}{1+m\exp\left[V_{ij}^{T}\gamma \right]}\right)\\ & \;\times \frac{1}{{\left[1+m+m\alpha \Lambda_{0}\left(t_{ij}\right)\exp\left[Z_{ij}^{T}\right]\right]}^{1+\frac{2}{\alpha }}}dm. \end{array}$$

Let, $\exp\left[V_{ij}^{T}\gamma \right]m=b$. Then $m=b\exp\left[-V_{ij}^{T}\gamma \right]$, and $dm=\exp\left[-V_{ij}^{T}\gamma \right]db$. This gives

$$\begin{array}{ll} \Psi & =\alpha^{\left(1+\frac{2}{\alpha }\right)}\int_{0}^{\infty }\left(\frac{{\left[\exp\left[-V_{ij}^{T}\gamma \right)b\right]}^{\left(1+\frac{1}{\alpha }\right)}}{1+b}\right)\\ & \frac{\exp\left[-V_{ij}^{T}\gamma \right]}{{\left[1+b\exp\left[-V_{ij}^{T}\gamma \right]+b\alpha \Lambda_{0}\left(t_{ij}\right)\exp\left[Z_{ij}^{T}\beta -V_{ij}^{T}\gamma \right]\right]}^{1+\frac{2}{\alpha }}}db, \end{array}$$

or

$$\begin{array}{cc} \Psi & =\exp\left[-V_{ij}^{T}\gamma \left(\frac{1}{\alpha }+2\right)\right]\alpha^{\left(1+\frac{2}{\alpha }\right)}\\ & \;\int_{0}^{\infty }\frac{b^{\left(1+\frac{1}{\alpha }\right)}}{\begin{array}{l} \left(1+b\right)\left[1+b\exp\left[-V_{ij}^{T}\gamma \right]\right.\\ {\left.+b\alpha \Lambda_{0}\left(t_{ij}\right)\exp\left[Z_{ij}^{T}\beta -V_{ij}^{T}\gamma \right]\right]}^{1+\frac{2}{\alpha }} \end{array}}db, \end{array}$$

Denoting $\Lambda_{0}\left(t_{ij}\right)\exp\left[Z_{ij}^{T}\beta -V_{ij}^{T}\gamma \right]=A$, an letting $b=\frac{d}{1-d}$ then have

$$\begin{array}{cc} \Psi & =\alpha^{1+\frac{2}{\alpha }}\exp\left[-V_{ij}^{T}\gamma \left(\frac{1}{\alpha }+2\right)\right]\\ & \;\int_{0}^{1}\frac{d^{1+\frac{1}{\alpha }}{\left(1-d\right)}^{-\left(1+\frac{1}{\alpha }\right)}}{\left(1+\frac{d}{1-d}\right)\cdot {\left[1+\frac{d}{1-d}\exp\left[-V_{ij}^{T}\gamma \right]+A\alpha \frac{d}{1-d}\right]}^{1+\frac{2}{\alpha }}}dd, \end{array}$$

or

$$\begin{array}{ll} \Psi & =\alpha^{1+\frac{2}{\alpha }}\cdot \exp\left[-V_{ij}^{T}\gamma \left(\frac{1}{\alpha }+2\right)\right]\\ & \;\int_{0}^{1}\frac{d^{\left(1+\frac{1}{\alpha }\right)}\cdot {\left(1-d\right)}^{\frac{1}{\alpha }-1}}{{\left[1-d\left(1-\exp\left[-V_{ij}^{T}\gamma \right]-A\alpha \right)\right]}^{1+\frac{2}{\alpha }}}dd. \end{array}$$

$$\begin{array}{ll} \Psi & =\alpha^{1+\frac{2}{\alpha }}\exp\left[-V_{ij}^{T}\gamma \left(\frac{1}{\alpha }+2\right)\right]\int_{0}^{1}d^{\frac{1}{\alpha }+2-1}\\ & \;{\left(1-d\right)}^{\left(\frac{1}{\alpha }-1\right)}\cdot {\left[1-d\left(1-\exp\left[-V_{ij}^{T}\gamma \right]-A\alpha \right)\right]}^{-\left(1+\frac{2}{\alpha }\right)}dd. \end{array}$$

However, the above integral has the form of a hyper‐geometric function that is, $F(a,b;c;z)=\frac{\Gamma \left(c\right)}{\Gamma \left(b\right)\Gamma \left(c-b\right)}\int_{0}^{1}t^{b-1}{\left(1-t\right)}^{c-b-1}{\left(1-zt\right)}^{-a}dt$. This gives the solution of the integral as

$$\begin{array}{ll} \Psi & =\frac{\alpha \Gamma \left(\frac{1}{\alpha }\right)}{\Gamma \left(\frac{2}{\alpha }+2\right)\Gamma \left(\frac{1}{\alpha }+2\right)}\exp\left[-V_{ij}^{T}\gamma \left(\frac{1}{\alpha }+2\right)\right]\\ & F\left(\left(1+\frac{2}{\alpha }\right),\left(2+\frac{1}{\alpha }\right);\left(2+\frac{2}{\alpha }\right);1-\exp\left[-V_{ij}^{T}\gamma \right]\right.\\ & \;\left.-\alpha \Lambda_{0}\left(t_{ij}\right)\exp\left[Z_{ij}^{T}\beta -V_{ij}^{T}\gamma \right]\right). \end{array}$$

No, putting back terms we have the marginal density function of t is

$$\begin{array}{ll} f(t_{ij}) & =\frac{\lambda_{0}\left(t_{ij}\right)\alpha \exp\left[-V_{ij}^{T}\gamma \left(\frac{1}{\alpha }+2\right)+Z_{ij}^{T}\beta \right]}{\Gamma \left(\frac{1}{\alpha }\right)\Gamma \left(2+\frac{2}{\alpha }\right)\Gamma \left(2+\frac{1}{\alpha }\right)}\\ & \;F\left(\left(1+\frac{2}{\alpha }\right),\left(2+\frac{1}{\alpha }\right);\left(2+\frac{2}{\alpha }\right);\right.\\ & \;\left.\left(1-\exp\left[-V_{ij}^{T}\gamma \right)-\alpha \Lambda_{0}\left(t_{ij}\right)\exp\left(Z_{ij}^{T}\beta -V_{ij}^{T}\gamma \right)\right)\right). \end{array}$$

#### Conditional Density Function $\omega_{1i}$ Given t

$$\begin{array}{lll} f\left(\omega_{2i}|t_{ij}\right)=\frac{\begin{array}{l} \left(\frac{2}{\alpha }+2\right)\exp\left[V_{ij}^{T}\gamma \left(\frac{1}{\alpha }+2\right)\right]\\ \exp\left[\omega_{2i}\left(1-\frac{1}{\alpha }\right)\right] \end{array}}{\begin{array}{l} B\left(\frac{1}{\alpha },\frac{1}{\alpha }\right)\left[\alpha \Lambda_{0}\left(t_{ij}\right)\exp\left[Z_{ij}^{T}\beta \right]\right.\\ {\left.+1+\exp\left[-\omega_{2i}\right]\right]}^{1+\frac{2}{\alpha }}\left(1+\exp\left[V_{ij}^{T}\gamma +\omega_{2i}\right]\right) \end{array}} & \; & \\ \times \frac{\Gamma \left(1+\frac{2}{\alpha }\right)\Gamma \left(2+\frac{1}{\alpha }\right)}{\begin{array}{l} F\left(\left(1+\frac{2}{\alpha }\right),\left(2+\frac{1}{\alpha }\right);\left(2+\frac{2}{\alpha }\right);\left(1-\exp\left[-V_{ij}^{T}\gamma \right]\right.\right.\\ -\;\left.\left.\alpha \Lambda_{0}\left(t_{ij}\right)\exp\left[Z_{ij}^{T}\beta -V_{ij}^{T}\gamma \right]\right)\right) \end{array}}. & & \end{array}$$

#### Conditional Density of $\omega_{1i}$ Given $\omega_{2}$ and t

The conditional density of $f\left(\omega_{1i}|t,\omega_{2i}\right)$ is obtained from $\frac{f\left(\omega_{1i},t,\omega_{2i}\right)}{f\left(t_{ij},\omega_{2i}\right)}$ which is

$$\begin{array}{ll} & f\left(\omega_{1i}|\omega_{2i},t_{ij}\right)=\frac{\begin{array}{l} \exp\left[\omega_{1i}\left(1+\frac{2}{\alpha }\right)\right]\exp\left[-\Lambda_{0}\left(t_{ij}\right)\exp\left(Z_{ij}^{T}\beta +\omega_{1i}\right)\right.\\ \left.-\frac{1}{\alpha }\left[\exp\left(\omega_{1i}\right)+\exp\left(\omega_{1i}-\omega_{2i}\right)\right]\right] \end{array}}{\begin{array}{l} \alpha^{1+\frac{2}{\alpha }}\Gamma \left(1+\frac{2}{\alpha }\right)\left[1+\exp\left(-\omega_{2i}\right)\right.\\ {\left.+\alpha \exp\left(Z_{ij}^{T}\beta \Lambda_{0}\left(t_{ij}\right)+\omega_{2i}\right)\right]}^{-\left(1+\frac{2}{\alpha }\right)} \end{array}}. \end{array}$$

#### Expectations of $\exp(-\omega_{2i}|t)$

$$\begin{array}{ll} & 𝔼\left[\exp\left(-\omega_{2i}|t_{ij}\right)\right]=\frac{\Gamma \left(2+\frac{1}{\alpha }\right)\exp\left[V_{ij}^{T}\gamma \right]}{\Gamma \left(\frac{1}{\alpha }\right)}\\ & \;\times \frac{\begin{array}{l} F\left(\left(1+\frac{2}{\alpha }\right),\left(1+\frac{1}{\alpha }\right);\left(2+\frac{2}{\alpha }\right);\right.\\ \left.\left(1-\exp\left(-V_{ij}^{T}\gamma \right)-\alpha \Lambda_{0}\left(t_{ij}\right)\exp\left(Z_{ij}^{T}\beta -V_{ij}^{T}\gamma \right)\right)\right) \end{array}}{\begin{array}{l} F\left(\left(1+\frac{2}{\alpha }\right),\left(2+\frac{1}{\alpha }\right);\left(2+\frac{2}{\alpha }\right);\right.\\ \left.\left(1-\exp\left(-V_{ij}^{T}\gamma \right)-\alpha \Lambda_{0}\left(t_{ij}\right)\exp\left(Z_{ij}^{T}\beta -V_{ij}^{T}\gamma \right)\right)\right) \end{array}}. \end{array}$$

#### Necessary Expectations

$$\begin{array}{ll} & 𝔼\left[\exp\left(\omega_{2i}|t_{ij}\right)\right]=\frac{\Gamma \left(2+\frac{1}{\alpha }\right)\Gamma \left(\frac{1}{\alpha }-1\right)\exp\left[-V_{ij}^{T}\gamma \right]}{\Gamma \left(\frac{1}{\alpha }\right)\cdot \Gamma \left(\frac{1}{\alpha }+3\right)}\\ & \times \frac{\begin{array}{l} F\left(\left(1+\frac{2}{\alpha }\right),\left(3+\frac{1}{\alpha }\right),\left(2+\frac{1}{\alpha }\right);\right.\\ \left.\left(1-\exp\left[-V_{ij}^{T}\gamma \right]-\alpha \Lambda_{0}\left(t_{ij}\right)\exp\left[Z_{ij}^{T}\beta -V_{ij}^{T}\gamma \right]\right)\right) \end{array}}{\begin{array}{l} F\left(\left(1+\frac{2}{\alpha }\right),\left(2+\frac{1}{\alpha }\right);\left(2+\frac{2}{\alpha }\right);\right.\\ \left.\left(1-\exp\left[-V_{ij}^{T}\gamma \right]-\alpha \Lambda_{0}\left(t_{ij}\right)\exp\left[Z_{ij}^{T}\beta -V_{ij}^{T}\gamma \right]\right)\right) \end{array}}. \end{array}$$

$$\begin{array}{ll} & 𝔼\left[\exp\left[\omega_{1i}\right]|\omega_{2i},t_{ij}\right]=\frac{\left(2+2\alpha \right)\exp\left[-V_{ij}^{T}\gamma \right]}{\left(3+\frac{2}{\alpha }\right)\left(\frac{1}{\alpha }+3\right)}\\ & \times \frac{\begin{array}{l} F\left(\left(2+\frac{2}{\alpha }\right),\left(3+\frac{1}{\alpha }\right),\left(3+\frac{2}{\alpha }\right);\right.\\ \left.\left(1-\exp\left[-V_{ij}^{T}\gamma \right]-\alpha \Lambda_{0}\left(t_{ij}\right)\exp\left[Z_{ij}^{T}\beta -V_{ij}^{T}\gamma \right]\right)\right) \end{array}}{\begin{array}{l} F\left(\left(1+\frac{2}{\alpha }\right),\left(2+\frac{1}{\alpha }\right),\left(2+\frac{2}{\alpha }\right);\right.\\ \left.\left(1-\exp\left[-V_{ij}^{T}\gamma \right]-\alpha \Lambda_{0}\left(t_{ij}\right)\exp\left[Z_{ij}^{T}\beta -V_{ij}^{T}\gamma \right]\right)\right) \end{array}}. \end{array}$$

$$\begin{array}{ll} & 𝔼[\exp[\omega_{1i}]\exp[-\omega_{2i}]|t]\\ & \;=\frac{\begin{array}{l} \alpha^{-\frac{2}{\alpha }-1}\left(2+\frac{2}{\alpha }\right)\left(1+\frac{1}{\alpha }\right)F\left(\left(2+\frac{2}{\alpha }\right),\left(1+\frac{2}{\alpha }\right),\left(2+\frac{3}{\alpha }\right);\right.\\ \left.\left(1-\exp\left[-V_{ij}^{T}\gamma \right]-\alpha \Lambda_{0}\left(t_{ij}\right)\exp\left[Z_{ij}^{T}\beta -V_{ij}^{T}\gamma \right]\right)\right) \end{array}}{\begin{array}{l} \left(3+\frac{2}{\alpha }\right)F\left(\left(1+\frac{2}{\alpha }\right),\left(2+\frac{1}{\alpha }\right),\left(2+\frac{2}{\alpha }\right);\right.\\ \left.\left(1-\exp\left[-V_{ij}^{T}\gamma \right]-\alpha \Lambda_{0}\left(t_{ij}\right)\exp\left[Z_{ij}^{T}\beta -V_{ij}^{T}\gamma \right]\right)\right) \end{array}}. \end{array}$$

Now $𝔼\left[\frac{1}{\left[1+\exp[V_{ij}^{T}\gamma +\omega_{2i}]\right]}|t_{ij}\right]=\int_{-\infty }^{\infty }\frac{f(\omega_{2i})d\omega_{2i}}{\left[1+\exp(V_{ij}^{T}\gamma +\omega_{2i})\right]}.$

$$\begin{array}{c} \;\Rightarrow \;\frac{\begin{array}{l} \Gamma \left(1+\frac{1}{\alpha }\right)F\left(\left(1+\frac{2}{\alpha }\right),\left(2+\frac{1}{\alpha }\right),\left(3+\frac{2}{\alpha }\right);\right.\\ \left.\left(1-\exp\left(-V_{ij}^{T}\gamma \right)-\alpha \Lambda \left(t_{ij}\right)\exp\left(Z_{ij}^{T}\beta -V_{ij}^{T}\gamma \right)\right)\right) \end{array}}{\begin{array}{l} \left(3+\frac{2}{\alpha }\right)F\left(\left(1+\frac{2}{\alpha }\right),\left(2+\frac{1}{\alpha }\right);\left(2+\frac{2}{\alpha }\right);\right.\\ \left.\left(1-\exp\left[-V_{ij}^{T}\gamma \right)-\alpha \Lambda_{0}\left(t_{ij}\right)\exp\left(Z_{ij}^{T}\beta -V_{ij}^{T}\gamma \right)\right)\right) \end{array}}. \end{array}$$

and

$$\begin{array}{ll} & 𝔼\;\left[\frac{1}{{\left[1+\exp[V_{ij}^{T}\gamma +\omega_{2i}]\right]}^{2}}|t_{ij}\right]=\frac{\Gamma \left(2+\frac{1}{\alpha }\right)\Gamma \left(2+\frac{2}{\alpha }\right)}{\Gamma \left(4+\frac{2}{\alpha }\right)\Gamma \left(\frac{1}{\alpha }\right)}\\ & \times \frac{\begin{array}{l} F\left(\left(1+\frac{2}{\alpha }\right),\left(2+\frac{1}{\alpha }\right),\left(4+\frac{2}{\alpha }\right);\right.\\ \left.\left(1-\exp\left(-V_{ij}^{T}\gamma \right)-\alpha \Lambda_{0}\left(t_{ij}\right)\exp\left(Z_{ij}^{T}\beta -V_{ij}^{T}\gamma \right)\right)\right) \end{array}}{\begin{array}{l} F\left(\left(1+\frac{2}{\alpha }\right),\left(2+\frac{1}{\alpha }\right);\left(2+\frac{2}{\alpha }\right);\right.\\ \left.\left(1-\exp\left[-V_{ij}^{T}\gamma \right)-\alpha \Lambda_{0}\left(t_{ij}\right)\exp\left(Z_{ij}^{T}\beta -V_{ij}^{T}\gamma \right)\right)\right) \end{array}}. \end{array}$$

$$\begin{array}{ll} & 𝔼\;\left[\frac{1}{{\left[1+\exp\left[V_{ij}^{T}\gamma +\omega_{2i}\right]\right]}^{3}}|t_{ij}\right]=\frac{\Gamma \left(2+\frac{1}{\alpha }\right)\Gamma \left(2+\frac{2}{\alpha }\right)\Gamma \left(3+\frac{1}{\alpha }\right)}{\Gamma \left(5+\frac{2}{\alpha }\right)\Gamma \left(\frac{1}{\alpha }\right)}\\ & \times \frac{\begin{array}{l} F\left(\left(1+\frac{2}{\alpha }\right),\left(2+\frac{1}{\alpha }\right),\left(5+\frac{2}{\alpha }\right);\right.\\ \left.\left(1-\exp\left(-V_{ij}^{T}\gamma \right)-\alpha \Lambda_{0}\left(t_{ij}\right)\exp\left(Z_{ij}^{T}\beta -V_{ij}^{T}\gamma \right)\right)\right) \end{array}}{\begin{array}{l} F\left(\left(1+\frac{2}{\alpha }\right),\left(2+\frac{1}{\alpha }\right);\left(2+\frac{2}{\alpha }\right);\right.\\ \left.\left(1-\exp\left[-V_{ij}^{T}\gamma \right)-\alpha \Lambda_{0}\left(t_{ij}\right)\exp\left(Z_{ij}^{T}\beta -V_{ij}^{T}\gamma \right)\right)\right) \end{array}}. \end{array}$$

### Identical Frailty

$$\begin{array}{cc} f\left(t_{ij}|u_{1i}\right) & =\lambda_{0}\left(t_{ij}\right)u_{1i}\exp\left[Z_{ij}^{T}\beta \right]\\ & \;\times \exp\left[-\Lambda \left(t_{ij}\right)\exp\left[Z_{ij}^{T}\beta \right]u_{1i}-\exp\left[V_{ij}^{T}\gamma \right]\right]. \end{array}$$

$$f\left(t_{ij}\right)=f\left(t_{ij}|u_{1i}\right)f\left(u_{i}\right)$$

$$f\left(t_{ij}\right)=\frac{\left(\alpha +1\right)\alpha^{\alpha }\lambda_{0}\left(t_{ij}\right)\exp\left[Z_{ij}^{T}\beta \right]}{{\left[\alpha +\exp\left[V_{ij}^{T}\gamma \right]+\Lambda_{0}\left(t_{ij}\right)\exp\left[Z_{ij}^{T}\beta \right]\right]}^{\alpha +1}}.$$

$$\begin{array}{cc} f\left(u_{1i}|t_{ij}\right) & =\frac{u_{1i}^{\alpha }\exp\left[-u_{1i}\left(\alpha +\exp\left[V_{ij}^{T}\gamma \right]+\Lambda_{0}\left(t_{ij}\right)\exp\left[Z_{ij}^{T}\beta \right]\right)\right]}{\Gamma \left(\alpha +1\right)}\\ & \;{\left[\alpha +\exp\left[V_{ij}^{T}\gamma \right]+\Lambda_{0}\left(t_{ij}\right)\exp\left[Z_{ij}^{T}\beta \right]\right]}^{\left(\alpha +1\right)}, \end{array}$$

and the expectations are

$$𝔼\left[u_{1i}|t_{ij}\right]=\frac{\alpha +2}{\left[\alpha +\exp\left[V_{ij}^{T}\gamma \right]+\Lambda_{0}\left(t_{ij}\right)\exp\left[Z_{ij}^{T}\beta \right]\right]}.$$

$$\begin{array}{cc} 𝔼\left[\log u_{1i}|t_{ij}\right] & =\psi \left(\alpha +1\right)-\ln\left[\alpha +\exp\left[V_{ij}^{T}\gamma \right]\right.\\ & \;+\left.\Lambda_{0}\left(t_{ij}\right)\exp\left[Z_{ij}^{T}\beta \right]\right]. \end{array}$$
