# Supplementary material for: Joint Frailty Mixture Cure Model for Recurrent Event Data With Dependent Censoring: An MCEM Approach
Source: Stat Med. 2026 May 8;45:e70579. doi: 10.1002/sim.70579 (PMC13155196; doi:10.1002/sim.70579)
Supplement: Supplementary file 1 — Data S1: Supporting Information. [file SIM-45-0-s001.pdf]

1 | SUPPLEMENTARY MATERIALS

1.1 | Densities and expectations of complementary log-log cure model.

The joint cumulant generating function of $\omega_{1i} = \log u_{1i}$ and $\omega_{2i} = \log u_{2i}$

$$\begin{aligned} k_{\omega_{1i},\omega_{2i}}(p,q) &= \log M_{\omega_{1i},\omega_{2i}}(p,q) \\ &= \log E\left[u_{1i}^p u_{2i}^q\right] \end{aligned}$$

Let, $x \sim Beta(\alpha,\alpha)$, $y \sim \Gamma(2\alpha,\alpha)$, and $u_{1i} = xy$. Therefore, we have $u_{1i} = xy \sim \Gamma(\alpha,\alpha)$ and $u_{2i} = 2y \sim \Gamma(2\alpha, 2\alpha)$. Now,

$$\begin{aligned} \mathbb{E}\left[u_{1i}^p u_{2i}^q\right] =& \mathbb{E}\left[(xy)^p, (2y)^q\right]. \\ &= 2^q \mathbb{E}\left[(x)^p, (y)^{p+q}\right]. \\ &= 2^q \mathbb{E}\left[(x)^p\right] \mathbb{E}\left[(y)^{p+q}\right]. \\ &= 2^q \frac{\Gamma(2\alpha)\Gamma(\alpha+p)}{\Gamma(\alpha)\Gamma(2\alpha+p)} \cdot \frac{\Gamma(2\alpha+p+q)}{\Gamma(2\alpha)\alpha^{p+q}} \end{aligned}$$

Now, the cumulant generating function is

$$\begin{aligned} k_{\omega_{1i},\omega_{2i}}(p,q) &= \log\left(2^q \cdot \frac{\Gamma(2\alpha)\Gamma(\alpha+p)}{\Gamma(\alpha)\Gamma(2\alpha+p)} \cdot \frac{\Gamma(2\alpha+p+q)}{\Gamma(2\alpha)\alpha^{p+q}}\right) \\ &= \log\Gamma(\alpha+p) - \log\Gamma(\alpha) - \log\Gamma(2\alpha+p) + \log\Gamma(2\alpha+p+q) + (p+q)\log\alpha + q\log 2 \end{aligned}$$

The mean, variance and covariance of ω_{1i} and ω_{2i} obtained using derivatives of cumulant generating function at $(p,q) = (0,0)$

$$\begin{aligned} \mathbb{E}(\omega_{1i}) &= \psi(\alpha) - \log\alpha, \\ \mathbb{E}(\omega_{2i}) &= \psi(2\alpha) - \log\alpha + \log 2, \\ \text{Var}(\omega_{1i}) &= \psi'(\alpha), \\ \text{Var}(\omega_{2i}) &= \psi'(2\alpha), \\ \text{Cov}(\omega_{1i},\omega_{2i}) &= \psi'(2\alpha), \\ \text{Corr}(\omega_{1i},\omega_{2i}) &= \frac{\psi'(2\alpha)}{\sqrt{\psi'(\alpha)}\sqrt{\psi'(2\alpha)}}, \end{aligned}$$

where $\psi(.)$ is the first derivative of the log gamma function and $\psi'(.)$ is the second derivative of it.

1.1.1 | Derivatives of log likelihood.

$$l_\gamma = \sum_i^N \sum_j^{n_i} \left(1 - U_{ij}\right) \log\left(1 - \pi_{ij}\right) + \sum_i^N \sum_j^{n_i} U_{ij} \log \pi_{ij} + \sum_i^N \sum_j^{n_i} \delta_{ij} \log \pi_{ij} - \sum_i^N \sum_j^{n_i} (1 - \delta_{ij})\pi_{ij}.$$

First derivative of l_γ, w.r.t. $\boldsymbol{\gamma}$, is

$$\begin{aligned} l'_\gamma = &\sum_i^N \sum_j^{n_i} \frac{\pi_{ij} \exp\left(\boldsymbol{V}_{ij}^T \gamma\right) u_{2i} V_{ij}}{1 - \pi_{ij}} - \sum_i^N \sum_j^{n_i} \delta_{ij} \exp\left[\boldsymbol{V}_{ij}^T \gamma\right] V_{ij} u_{2i} + \sum_i^N \sum_j^{n_i} \delta_{ij} \pi_{ij} \exp\left[\boldsymbol{V}_{ij}^T \gamma\right] V_{ij} u_{2i} \\ &- \sum_i^N \sum_j^{n_i} \frac{2\alpha^{2\alpha} u_{2i}^{2\alpha} \exp\left[-2\alpha u_{2i}\right]}{\Gamma 2\alpha} \pi_{ij} V_{ij} \exp\left[\boldsymbol{V}_{ij}^T \gamma\right] \left[1 - \exp\left(\boldsymbol{V}_{ij}^T \gamma\right) u_{2i}\right] - \\ &\sum_i^N \sum_j^{n_i} \frac{[2\alpha]^{2\alpha} u_{2i}^{2\alpha} \exp\left[\boldsymbol{V}_{ij}^T \gamma\right] V_{ij}}{\Gamma 2\alpha} \left(\frac{\exp\left(-2u_{2i}\alpha\right) \pi_{ij} \pi_{ij}}{1 - \pi_{ij}} - \log\left[1 - \pi_{ij}\right] \pi_{ij} \exp\left[-u_{2i} 2\alpha\right]\right). \end{aligned}$$

Second derivative of l_γ is

$$\begin{aligned} l''_\gamma = &\sum_i^N \sum_j^{n_i} u_{2i} V^T V \left(\frac{\pi_{ij} \exp\left(\boldsymbol{V}_{ij}^T \gamma\right)}{1 - \pi_{ij}} - \frac{\pi_{ij}^2 \exp\left(2\boldsymbol{V}_{ij}^T \gamma\right) u_{2i}}{\left[1 - \pi_{ij}\right]^2} - \frac{\pi_{ij} \exp\left(2\boldsymbol{V}_{ij}^T \gamma\right) u_{2i}}{\left[1 - \pi_{ij}\right]}\right) - \sum_i^N \sum_j^{n_i} \frac{[2\alpha]^{2\alpha} u_{2i}^{2\alpha} \exp\left[-2u_{2i}\alpha\right] V^T V}{\Gamma 2\alpha} \\ &\left(\frac{\pi_{ij} \pi_{ij} \exp\left(\boldsymbol{V}_{ij}^T \gamma\right)}{1 - \pi_{ij}} - \frac{2u_{2i} V_{ij} \pi_{ij} \exp\left(2\boldsymbol{V}_{ij}^T \gamma\right)}{1 - \pi_{ij}}\right) \\ &- \sum_i^N \sum_j^{n_i} \frac{[2\alpha]^{2\alpha} u_{2i}^{2\alpha} \exp\left[-2u_{2i}\alpha\right] V^T V}{\Gamma 2\alpha} \left(\frac{u_{2i} \pi_{ij}^3 \exp\left(2\boldsymbol{V}_{ij}^T \gamma\right)}{\left[1 - \pi_{ij}\right]^2} - \frac{u_{2i} \pi_{ij}^2 \exp\left(2\boldsymbol{V}_{ij}^T \gamma\right)}{1 - \pi_{ij}}\right) \end{aligned}$$

$$l_\beta(\beta) = \sum_i^N \sum_j^{n_i} \delta_{ij} \log\left(\lambda_0\left(t_{ij}\right)\right) + \sum_i^N \sum_j^{n_i} \delta_{ij}\left(\boldsymbol{Z}_{ij}^T\beta + \omega_{1i}\right) - \sum_i^N \sum_j^{n_i} \Lambda_0\left(t_{ij}\right) \exp\left[\boldsymbol{Z}_{ij}^T\beta + \omega_{1i}\right] + \sum_i^N \sum_j^{n_i} (1-\delta_{ij}) \exp\left[-\Lambda_0\left(t_{ij}\right) \exp\left[\boldsymbol{Z}_{ij}^T\beta\right] + \omega_{1i}\right] - \exp\left[\boldsymbol{V}_{ij}^T\hat{\gamma}\right].$$

The first derivative of expected l_β is

$$\mathbb{E}\left(l'_\beta\right) = \sum_i^N \sum_j^{n_i} Z_{ij}\delta_{ij} - \sum_i^N \sum_j^{n_i} \delta_{ij}\Lambda_0\left(t_{ij}\right) \mathbb{E}\left[u_{1i}|t_{ij}\right] Z_{ij} \exp\left[\boldsymbol{Z}_{ij}^T\beta\right] - \sum_i^N \sum_j^{n_i} (1-\delta_{ij}) \left[\Lambda_0\left(t_{ij}\right) \mathbb{E}\left[u_{1i}|t_{ij}\right] \exp\left(\boldsymbol{Z}_{ij}^T\beta\right) Z_{ij} \mathbb{E}\left[\exp\left[-\Lambda_0\left(t_{ij}\right) \exp\left[\boldsymbol{Z}_{ij}^T\beta\right] u_{1i}\right]\right] |t_{ij}\right] \exp\left[\boldsymbol{V}_{ij}^T\hat{\gamma}\right].$$

The second derivative of expected l_β is

$$\mathbb{E}\left(l''_\beta\right) = -\sum_i^N \sum_j^{n_i} (1-\delta_{ij}) \boldsymbol{Z}_{ij}^T \boldsymbol{Z_{ij}} \Lambda_0\left(t_{ij}\right) \exp\left[\boldsymbol{V}_{ij}^T\hat{\gamma}\right] \mathbb{E}\left[u_{1i}|t_{ij}\right] \exp\left[\boldsymbol{Z_{ij}}^T\beta\right]$$

$$\mathbb{E}\left[\exp\left[-\Lambda_0\left(t_{ij}\right) \exp\left[\boldsymbol{Z}_{ij}^T\beta\right] u_{1i}\right] |t_{ij}\right] \left(1 + \Lambda_0\left(t_{ij}\right) \mathbb{E}\left[u_{1i}|t_{ij}\right] \exp\left[\boldsymbol{Z_{ij}}^T\beta\right]\right) - \sum_i^N \sum_j^{n_i} \delta_{ij}\Lambda_0\left(t_{ij}\right) E\left[u_{1,i}|t_{i,j}\right] \exp\left[\boldsymbol{Z_{ij}}^T\beta\right] \boldsymbol{Z}_{ij}^T \boldsymbol{Z}_{ij}.$$

$$l_\alpha = 2N\alpha\log\alpha - \alpha N\log 2 - 2N\log\Gamma(\alpha) - \frac{\alpha}{2}\sum_i^N u_{2i} + \alpha\sum_i^N \log u_{1i} - (\alpha-1)\sum_i^N \log(u_{2i} - 2u_{1i}).$$

First derivative of expected l_α is

$$\mathbb{E}\left(l'_\alpha\right) = 2N + 2N\log\alpha - N\log 2 - 0.5\sum_i^N \mathbb{E}\left[u_{2i}|t_{i.}\right] + \sum_i^N \mathbb{E}\left[\log u_{1i}|t_{i.}\right] + \sum_i^N \mathbb{E}\left[\log(u_{2i} - 2u_{1i})|t_{i.}\right] - 2N\psi(\alpha).$$

and second derivative of l_α is

$$l''_\alpha = \frac{2N}{\alpha} - 2N\psi'(\alpha).$$

1.1.2 | Expectations of frailty associated with hazard model given each time.

The expectations of u_{1i} given each individual recurrence time, t_{ij}, is obtained by following steps:

- Step 1: $f\left(t_{ij}|u_i\right) = \frac{f\left(t_{ij},u_{1i}\right)}{f\left(u_{ij}\right)}$ with

$$f\left(t_{ij}|u_i\right) = \frac{\left(\frac{\alpha}{2}\right)^\alpha \lambda_0\left(t_{ij}\right) \exp\left[\boldsymbol{Z}_{ij}^T\beta\right] u_{1i}{}^\alpha}{\left[\exp\left[\boldsymbol{V}_{ij}^T\gamma\right] + \frac{\alpha}{2}\right]} \exp\left[-u_{1i}\left(\Lambda_0\left(t_{ij}\right) \exp\left[\boldsymbol{Z}_{ij}^T\beta\right] + 2\exp\left[\boldsymbol{V}_{ij}^T\gamma\right]\right)\right].$$

- Step 2: The conditional density of total survival time during the follow-up $f\left(t_{i.}|u_{1i}\right) = \prod_j^{n_i} f\left(t_{ij}|u_i\right)$.

$$f\left(t_{i.}|u_{1i}\right) = \left(\frac{\alpha}{2}\right)^\alpha \prod_j^{n_i} \lambda_0\left(t_{ij}\right) \exp\left[\sum_j^{n_i} \boldsymbol{Z}_{ij}^T\beta\right] u_{1i} \prod_j^{n_i} \left[\exp\left[\boldsymbol{V}_{ij}^T\gamma\right] + \frac{\alpha}{2}\right]^{-\alpha} \exp\left[-u_{1i}\left(\Lambda_0\left(t_{ij}\right) \sum_j^{n_i} \exp\left[\boldsymbol{Z}_{ij}^T\beta\right] + 2\sum_j^{n_i} \exp\left[\boldsymbol{V}_{ij}^T\gamma\right]\right)\right].$$

- Step 3: Determine joint density of $f(t_{i.}, u_{1i}) = f(t_{i.}|u_{1i})f(u_{1i})$. That is,

$$f(t_{i.}, u_{1i}) = \left(\frac{\alpha}{2}\right)^{\alpha} \alpha^{\alpha} \prod_{j}^{n_i} \lambda_0(t_{ij}) \exp\left[\sum_{j}^{n_i} \boldsymbol{Z}_{ij}^{T}\beta\right] u_{1i}^{\alpha} \prod_{j}^{n_i}\left[\exp\left[\boldsymbol{V}_{ij}^{T}\gamma\right] + \frac{\alpha}{2}\right]^{-\alpha} \exp\left[-u_{1i}\left(\alpha + 2\sum_{j}^{n_i}\exp\left[\boldsymbol{V}_{ij}^{T}\gamma + \Lambda_0(t_{ij})\sum_{j}^{n_i}\exp\left[\boldsymbol{Z}_{ij}^{T}\beta\right]\right]\right)\right].$$

- Step 4: Conditional density of $f(u_{1i}|t_{i.}) = \frac{f(t_{i.}, u_{1i})}{f(t_{i.})}$ where $f(t_{i.}) = \int_{u_1} f(u_{1i}, t_{i.})\, du_{1i}$. That is,

$$f(t_{i.}) = \frac{(\alpha+1)\alpha^{2\alpha}2^{-\alpha}\prod_{j}^{n_i}\lambda_0(t_{ij})\exp\left[\sum_{j}^{n_i}\boldsymbol{Z}_{ij}^{T}\beta\right]\prod_{j}^{n_i}\left[\exp\left[\boldsymbol{V}_{ij}^{T}\gamma\right] + \frac{\alpha}{2}\right]^{-\alpha}}{\prod_{j}^{n_i}\left[\alpha + 2\sum_{j}^{n_i}\exp\left[\boldsymbol{V}_{ij}^{T}\gamma\right] + \sum_{j}^{n_i}\Lambda_0(t_{ij})\exp\left[\boldsymbol{Z}_{ij}^{T}\beta\right]\right]^{\alpha+1}}.$$

$$f(u_{1i}|t_{i.}) = \frac{u_{1i}^{\alpha}\exp\left[-u_{1i}\left(\alpha + 2\sum_{j}^{n_i}\exp\left[\boldsymbol{V}_{ij}^{T}\gamma\right] + \Lambda_0(t_{ij})\sum_{j}^{n_i}\exp\left[\boldsymbol{Z}_{ij}^{T}\beta\right]\right)\right]}{(\alpha+1)\Gamma\alpha\left[\alpha + 2\sum_{j}^{n_i}\exp\left[\boldsymbol{V}_{ij}^{T}\gamma\right] + \Lambda_0(t_{ij})\sum_{j}^{n_i}\exp\left[\boldsymbol{Z}_{ij}^{T}\beta\right]\right]^{-(\alpha+1)}}.$$

- Step 5:

$$\mathbb{E}\left[u_{1i}|t_{i.}\right] = \frac{\alpha}{\left[\alpha + 2\sum_{j}^{n_i}\exp\left[\boldsymbol{V}_{ij}^{T}\gamma\right] + \sum_{j}^{n_i}\Lambda_0(t_{ij})\exp\left[\boldsymbol{Z}_{ij}^{T}\beta\right]\right]}.$$

1.1.3 | Density and expectation of frailty associated with complementary log-log component given frailty associated with hazard model and each recurrence time.

Joint density of $f(u_{1i}, u_{2j}, t_{ij})$ is obtained by $f(u_{1i}, u_{2j}, t_{ij}) = f(t_{ij}|u_{1i}, u_{2j})f(u_{1i}, u_{2i})$ which is given by

$$f\left(u_{1i}, u_{2i} \mid t_{ij}\right) = \frac{1}{(\alpha+1)\,\Gamma(\alpha)^2}\left(\exp[\boldsymbol{V_{ij}}^{T}\gamma] + \frac{\alpha}{2}\right)^{\alpha}\left(\alpha + 2\exp[\boldsymbol{V_{ij}}]^{T}\gamma + \Lambda_0(t_{ij})\exp[\boldsymbol{Z}_{ij}{}^{T}\beta\right)^{\alpha+1} \times \exp\left[-u_{2i}\left(\exp[\boldsymbol{V_{ij}}^{T}\gamma] + \tfrac{\alpha}{2}\right) - u_{1i}\,\Lambda_0(t_{ij})\exp[\boldsymbol{Z_{ij}}^{T}\beta]\right] u_{1i}^{\alpha}\left(u_{2i} - 2u_{1i}\right)^{\alpha-1},$$

$$f\left(u_{1i}, u_{2j}, t_{ij}\right) = \frac{2^{-\alpha}\alpha^{2\alpha}\lambda_0(t_{ij})\exp\left[\boldsymbol{Z}_{ij}^{T}\beta\right]}{\Gamma(\alpha)^2}\exp\left[-u_{2i}\exp\left[\boldsymbol{V}_{ij}^{T}\gamma\right] - u_{1i}\Lambda_0(t_{ij})\exp\left[\boldsymbol{Z}_{ij}^{T}\beta\right]\right]\exp\left[-\frac{\alpha u_{2i}}{2}\right]u_1{}^{\alpha}[u_{2i} - 2u_{1i}]^{\alpha-1}.$$

Conditional density of $f\left(u_{2i}|u_{1i}, t_{ij}\right) = \frac{f\left(u_{1i}, u_{2j}, t_{ij}\right)}{f\left(u_{1i}, t_{ij}\right)}$ is given by

$$f\left(u_{2i}|u_{1i}, t_{ij}\right) = \frac{\left[\exp \boldsymbol{V}_{ij}{}^{T}\gamma + \frac{\alpha}{2}\right]^{\alpha}}{\Gamma(\alpha)}(u_{2i} - 2u_{1i})^{\alpha-1}\exp\left[-u_{2i}\left(\exp\left[\boldsymbol{V}_{ij}^{T}\gamma\right] + \frac{\alpha}{2}\right)\right]\exp\left[u_{1i}\left(2\exp\left[\boldsymbol{V}_{ij}^{T}\gamma\right] + \alpha\right)\right].$$

The expectation of $u_{2i}|u_{1i}, t_{ij}$ is

$$\mathbb{E}\left(u_{2i}|u_{1i}, t_{ij}\right) = \frac{\alpha}{\left[\frac{\alpha}{2} + \exp\left[\boldsymbol{V}_{ij}^{T}\gamma\right]\right]} + 2\mathbb{E}\left[u_{1i}|t_{i.}\right].$$

1.1.4 | Expectation of being cure given data.

$$\mathbb{E}\left[U|t_{ij} > t, u_{2i}\right] = \frac{P\left(U_{ij} = 1\right)S\left(t_{ij}|U_{ij} = 1\right)}{1 - P\left(U_{ij} = 1\right) + P\left(U_{ij} = 1\right)S\left(t_{ij}|U_{ij} = 1\right)},$$

where

$$P\left[U = 1|u_{2i}, t_{ij}\right] = \frac{[2\alpha]^{2\alpha}\exp\left[-u_{2i}\left[\exp\left[\boldsymbol{V}_{ij}^{T}\gamma\right] + 2\alpha\right]\right]u_{2i}^{2\alpha-1}}{\Gamma(2\alpha)},$$

and

$$S\left(t_{ij}|U_{ij}=1\right)=\int_0^{\infty} S\left(t_{ij}|U_{ij}=1,u_1\right)f(u_1)\,du_1=\int_0^{\infty}\exp[-\Lambda_0(t_{ij})\exp\left[\boldsymbol{Z}_{ij}^T\beta\right]u_1]f(u_1)\,du_1$$
$$=\left[\frac{\alpha}{\alpha+\Lambda_0(t_{ij})\exp\left[\boldsymbol{Z}_{ij}^T\beta\right]}\right]^{\alpha}.$$

1.1.5 | Expectations of frailty associated with complementary log-log component given each time.

$$\mathbb{E}\left[u_{2i}|t_{ij}\right]=\int_{u_1}\mathbb{E}\left(u_{2i}|u_{1i},t_{ij}\right)f\left(u_{1i}|t_{ij}\right)du_1.$$

$$\mathbb{E}\left[u_{2i}|t_{ij}\right]=\frac{\alpha}{\left[\frac{\alpha}{2}+\exp\left[\boldsymbol{V}_{ij}^T\gamma\right]\right]}+\frac{2\left(\alpha+1\right)}{\left[\alpha+2\exp\left[\boldsymbol{V}_{ij}^T\gamma\right]+\Lambda_0\left(t_{ij}\right)\exp\left[\boldsymbol{Z}_{ij}^T\beta\right]\right]}.$$

Therefore, the $\mathbb{E}\left[u_{2i}|t_{i.}\right]$ is derived as

$$\mathbb{E}\left[u_{2i}|t_{i.}\right]=\frac{\alpha}{\prod_j^{n_i}\left[\frac{\alpha}{2}+\exp\left[\boldsymbol{V}_{ij}^T\gamma\right]\right]}+\frac{2\left(\alpha+1\right)}{\left[\alpha+2\sum_j^{n_i}\exp\left[\boldsymbol{V}_{ij}^T\gamma\right]+\sum_j^{n_i}\Lambda_0\left(t_{ij}\right)\exp\left[\boldsymbol{Z}_{ij}^T\beta\right]\right]}.$$

1.1.6 | Necessary expectations of the components of the complete data log likelihood for EM algorithm.

$$\mathbb{E}\left[\log u_{1i}|t_{ij}\right]=\psi\left(\alpha+1\right)-\ln\left[\alpha+2\exp\left(\boldsymbol{V}_{ij}^T\gamma\right)+\Lambda_0\left(t_{ij}\right)\exp\left(\boldsymbol{Z}_{ij}^T\beta\right)\right].$$

$$\mathbb{E}\left[\log u_{1i}|t_{i.}\right]=\psi\left(\alpha+1\right)-\ln\left[\alpha+2\sum_j\exp\left(\boldsymbol{V}_{ij}^T\gamma\right)+\sum_j\Lambda_0\left(t_{ij}\right)\exp\left(\boldsymbol{Z}_{ij}^T\beta\right)\right].$$

$$\mathbb{E}\left[u_{2i}-2u_{1i}|t_{ij}\right]=\psi\left(\alpha\right)-\ln\left[\exp\left(\boldsymbol{V}_{ij}^T\gamma\right)+\frac{\alpha}{2}\right],\text{where}\quad \psi_{u_{2i}-2u_{1i}}\left(\alpha\right)=\frac{d\ln\Gamma\left(\alpha\right)}{x}.$$

Therefore,

$$\mathbb{E}\left[u_{2i}-2u_{1i}|t_{i.}\right]=\psi\left(\alpha\right)-\ln\left[\sum_j\exp\left(\boldsymbol{V}_{ij}^T\gamma\right)+\frac{\alpha}{2}\right].$$

$$\mathbb{E}\left[\log u_{2i}|t_{i.}\right]\approx\log\mathbb{E}\left[u_{2i}|t_{i.}\right]-\frac{V\left[u_{2i}|t_{i.}\right]}{2\mathbb{E}\left[u_{2i}^2|t_{i.}\right]}=\log\mathbb{E}\left[u_{2i}|t_{i.}\right]-\frac{1}{2}+\frac{\mathbb{E}\left[u_{2i}|t_{i.}\right]^2}{2\mathbb{E}\left[u_{2i}^2|t_{i.}\right]}.$$

$$\mathbb{E}\left[u_{2i}^2|t_{i.}\right]=\frac{\left(\alpha+2\right)}{\left(\alpha+1\right)\prod_j^{n_i}\left[\frac{\alpha}{2}+\exp\left[\boldsymbol{V}_{ij}^T\gamma\right]\right]^2}+\frac{4\left(\alpha+1\right)\left(\alpha+2\right)}{\prod_j^{n_i}\left[\frac{\alpha}{2}+\exp\left[\boldsymbol{V}_{ij}^T\gamma\right]\right]\left[\alpha+2\sum_j^{n_i}\exp\left[\boldsymbol{V}_{ij}^T\gamma\right]+\sum_j^{n_i}\Lambda_0\left(t_{ij}\right)\exp\left[\boldsymbol{Z}_{ij}^T\beta\right]\right]}$$
$$+\frac{4\left(\alpha+2\right)\left(\alpha+3\right)}{\left[\alpha+2\sum_j^{n_i}\exp\left[\boldsymbol{V}_{ij}^T\gamma\right]+\sum_j^{n_i}\Lambda_0\left(t_{ij}\right)\exp\left[\boldsymbol{Z}_{ij}^T\beta\right]\right]^2}.$$

1.2 | Correlation of frailties of logistic cure model.

Consider the frailty components

$$\omega_1 = \log(u_1 u_2) = \log u_1 + \log u_2, \qquad \omega_2 = \log \frac{u_2}{1-u_2} = \log u_2 - \log(1-u_2),$$

where $u_1 \sim \Gamma\left(\frac{2}{\alpha}, \frac{1}{\alpha}\right)$ and $u_2 \sim \text{Beta}\left(\frac{1}{\alpha}, \frac{1}{\alpha}\right)$ independently. Using standard results for logarithmic moments of Gamma and Beta distributions, we have

$$\text{Var}(\log u_1) = \psi'\left(\frac{2}{\alpha}\right), \quad \text{Var}(\log u_2) = \psi'\left(\frac{1}{\alpha}\right) - \psi'\left(\frac{2}{\alpha}\right), \quad \text{Cov}(\log u_2, \log(1-u_2)) = -\psi'\left(\frac{2}{\alpha}\right),$$

where $\psi'(\cdot)$ is the trigamma function. From these, the variance of the frailties are

$$\text{Var}(\omega_1) = \text{Var}(\log u_1) + \text{Var}(\log u_2) = \psi'\left(\frac{1}{\alpha}\right), \qquad \text{Var}(\omega_2) = \text{Var}(\log u_2) + \text{Var}(\log(1-u_2)) - 2\,\text{Cov}(\log u_2, \log(1-u_2)) = 2\,\psi'\left(\frac{1}{\alpha}\right),$$

and the covariance

$$\text{Cov}(\omega_1, \omega_2) = \text{Var}(\log u_2) - \text{Cov}(\log u_2, \log(1-u_2)) = \psi'\left(\frac{1}{\alpha}\right).$$

Hence, the correlation coefficient between ω_1 and ω_2 is

$$\rho(\omega_1, \omega_2) = \frac{\text{Cov}(\omega_1, \omega_2)}{\sqrt{\text{Var}(\omega_1)\text{Var}(\omega_2)}} = \frac{\psi'(1/\alpha)}{\sqrt{\psi'(1/\alpha) \cdot 2\psi'(1/\alpha)}} = \frac{1}{\sqrt{2}}.$$
